# Safe-by-redesign guidance for toxic industrial chemicals using explainable artificial intelligence: Introducing the DETOX-QSAR model

**DOI:** 10.1038/s41598-026-48176-0

**Published:** 2026-04-18

**Authors:** Feyza Kelleci̇ Çeli̇k, Seyyide Doğan

**Affiliations:** 1 Karamanoğlu Mehmetbey University, Vocational School of Health Services, Department of Anesthesia, Karaman, 70200 Turkey; 2 Karamanoğlu Mehmetbey University, Faculty of Economics and Administrative Science, Management Information System, Karaman, 70200 Turkey

**Keywords:** Data-driven, SHapley additive exPlanations, Toxicoinformatics, Toxic industrial chemicals, Green toxicology, Computational toxicology, Chemistry, Computational biology and bioinformatics, Drug discovery, Environmental sciences

## Abstract

Toxic industrial chemicals (TICs) are dual-use agents with potential for immediate and lethal inhalation toxicity upon exposure. This study first introduced the Descriptor-Engineered Transition to Non-Toxicity via QSAR (DETOX-QSAR) model, a tool for predicting and designing out the acute inhalation toxicity of TICs. We aimed to proactively prevent adverse effects by addressing safety concerns early in the molecular design phase. Inhalation toxicity was modeled using Support Vector Machine (SVM), Random Forest (RF), Extreme Gradient Boosting (XGBoost), and Categorical Boosting (CatBoost) via consensus feature engineering. SHapley Additive exPlanations (SHAP) findings indicate that nX and minwHBa play a decisive role in prediction. Arsine was identified as the highest-risk pilot compound via Multi-Criteria Decision Making (MCDM) for the modification process. Guided by the local SHAP method, descriptor values of Arsine were gradually modified until toxicity was eliminated. This explainable artificial intelligence (XAI) approach advances green toxicology, enhancing international security against the growing chemical industry.

**Supplementary Information:**

The online version contains supplementary material available at 10.1038/s41598-026-48176-0.

## Main

Industrial chemicals are legally produced, distributed, transported, and stored for commercial purposes. Despite being essential components of the global economy, industrial chemicals labeled as toxic pose serious risks to health and the environment, potentially leading to mass casualties. Toxic industrial chemicals (TICs) are high-concern substances with lethal concentration of 50 (LCt_50_) values below 100.000 mg-min/m³ by inhalation in any mammal^1^. The primary target of an acute exposure to TICs is the respiratory system. They can induce lethal respiratory toxicity following short-term inhalation due to their rapid and irreversible effects. TICs may be released - accidentally, intentionally, or due to natural disasters - at any location and time, potentially causing catastrophic consequences^2^. According to the North Atlantic Treaty Organization (NATO)’s policy on weapons of mass destruction, TICs pose security risks due to their potential for weaponization^3^. They can be an obvious choice for terrorist attacks or environmental sabotage because of their inherent toxicity, large-scale production, low cost, and legal accessibility^4^. Compared to traditional chemical warfare agents (CWAs), weaponized industrial chemicals can produce higher-consequence hazard scenarios depending on their quantity; therefore, effective management of TIC risk is crucial^1^.

Artificial intelligence (AI)-supported computational modalities, such as quantitative structure-activity relationship (QSAR) models, can serve as a component of risk assessment. They are rapid, ethical, and cost-effective solutions for identifying the toxicity profiles of chemicals^5,6^. Although several environmental agencies have adopted in silico techniques^7^, QSAR models specifically targeting TICs remain limited. To address this gap, we introduce the Descriptor-Engineered Transition to Non-Toxicity (DETOX) via QSAR to predict and prevent acute inhalation toxicity risks of TICs. Beyond predicting toxicity, DETOX-QSAR can assist in developing safe-by-redesign strategies. In this study, the safe-by-redesign framework is not intended to represent a synthesis scheme for direct chemical transformations; rather, it is presented as a design guidance framework that elucidates descriptor-level trends associated with toxicity as learned by the QSAR model. The gradual modification of descriptors does not directly correspond to discrete structural modifications. Instead, the modification step provides an analytical approach for quantitatively identifying the direction and relative magnitude of descriptor changes associated with reduced toxicity, rather than directly proposing synthesizable molecules. Overall, this approach is positioned as a descriptor-engineering framework that informs, constrains, and guides model-based optimization efforts. Our binary models were developed using toxic (available TICs)^2^ and safe chemicals^8^ for the inhalation route. Machine learning (ML) models were developed using selected descriptors, and the insights behind their predictions were explained through the SHapley Additive exPlanations (SHAP) method^9^. Critical descriptors contributing to the target effect were presented compound-by-compound using local SHAP explanations. The highest-risk chemical in the test set was then identified through a layered selection process, and its descriptors were optimized to improve its safety profile. In this process, candidate compounds were derived using grid optimization on critical descriptors based on SHAP contribution values, re-evaluating toxicity risks until safe alternatives were obtained. This approach aims to set safe boundaries for AI-driven descriptor modification before advancing to chemical synthesis. The stepwise modification framework with QSAR, developed for the selected pilot chemical, can be applied to any labeled or unlabeled compound to evaluate and prevent acute inhalation toxicity. Incorporating DETOX-QSAR models early in molecular design helps avoid toxicity by guiding structural changes. Furthermore, the model’s real-world applications include categorizing and labeling TICs, enabling rapid hazard identification.

This model was developed to predict the immediate inhalation risk associated with TICs and to prevent such risks at the molecular level. In this study, green toxicology principles were applied through structural optimizations guided by the DETOX-QSAR method. By using safer values of molecular identifier, we aimed to design out potential toxicity from the early stages of chemical research and development (R&D). Unlike empirical trial-and-error methods, our model offers rational and targeted chemical design using in silico approaches. We propose a benign descriptor design strategy that optimizes molecular descriptor values to support safe-by-redesign principles. The XAI-based study contributes to proactive risk management of TICs by helping to identify toxic substances and prevent possible risks.

## Results

### Descriptor-based outcomes

#### Calculation of the features

Descriptor calculation and preprocessing were performed to encode chemical structures into numerical features suitable for DETOX-QSAR modeling. We calculated 1444 two-dimensional (2D) descriptors for each chemical using the open-source PaDEL software^10^.

#### Consensus feature selection strategy

In the proposed framework, feature selection is approached as a consensus-driven strategy rather than a single-method identification process. Accordingly, a consensus molecular descriptor subset was constructed based on agreement across multiple feature selection techniques. This consensus approach, hereafter referred to as meta-descriptor, employs a plurality voting scheme to retain descriptors that are consistently supported by various selection mechanisms. The underlying rationale is that individual feature selection methods capture complementary aspects of the underlying structure–property relationships; therefore, their aggregation enhances model robustness and predictive reliability^11,12^.

Table 1 presents the parameters of all feature selection methods (Lasso, Elastic Net, Random Forest, Random Forest-Recursive Feature Elimination, Consensus), the corresponding number of selected molecular descriptors, and the mean prediction accuracies obtained from 30 repeated results. These analyses were conducted exclusively as a preliminary, decision-enabling assessment to compare the effectiveness and stability of different feature selection strategies under identical conditions. Support Vector Machine (SVM) was adopted in this stage due to its computational efficiency and consistently robust predictive performance.

**Table 1:** Prediction results of the chosen model for different feature selection methods.

Methods	Feature Count (*n*)	Accuracy mean (±S.D.)
Non Selection	1444	0.8561 (±0.0397)
LASSO (max iteration:10000)	68	0.8695 (±0.0304)
ElNet (max iteration:10000; l1 ratio:0.1)	124	0.8842 (±0.0311)
RF (max depth: 15; n. of estimator: 1000)	100	0.8760 (±0.0398)
RF-RFE (estimator=RF; n.of select feature=100)	100	0.8619 (±0.0373)
Consensus (Receiving votes from three out of four methods)	21	0.8912 (±0.03668)

LASSO: Least Absolute Shrinkage and Selection Operator; ElNet: Elastic Net Regularization Scheme; RF: Random Forest; RFE: Recursive Feature Elimination; S.D.: Standard Deviation.

After confirming normality (Shapiro-Wilk Test: 0.9602, *p*-value = 0.3128), analysis of variance revealed among feature selection strategies (ANOVA test statistic: 4.0767, *p*-value: 0.00159). Post-hoc Tukey HSD analysis indicated that the consensus strategy and the Elastic Net regularization scheme significantly outperformed the non-selection baseline (mean diff: 0.0351, *p*-value: 0.003 and mean diff: 0.0281, *p*-value: 0.0346, respectively). Furthermore, the consensus approach yielded significantly better performance than the Random Forest–Recursive Feature Elimination (RF-RFE) method (mean diff: 0.0292, *p*-value: 0.024).

Based on this statistical evidence, the consensus feature selection strategy was identified as the most stable and reliable approach. Importantly, the specific descriptor subsets obtained in these preliminary experiments were not propagated to the final modeling stage. Instead, only the validated selection strategy itself was retained and subsequently embedded within the nested cross-validation framework of the main modeling pipeline.

During main model development, the consensus selection strategy was re-applied independently within each training fold of the nested cross-validation procedure, resulting in fold-specific optimal feature subsets. Selection stability was evaluated across folds, and descriptors that were consistently selected (i.e., observed in at least two folds) were subsequently retained to construct a stable feature representation for downstream modeling and external validation.

**Fig. 1:**
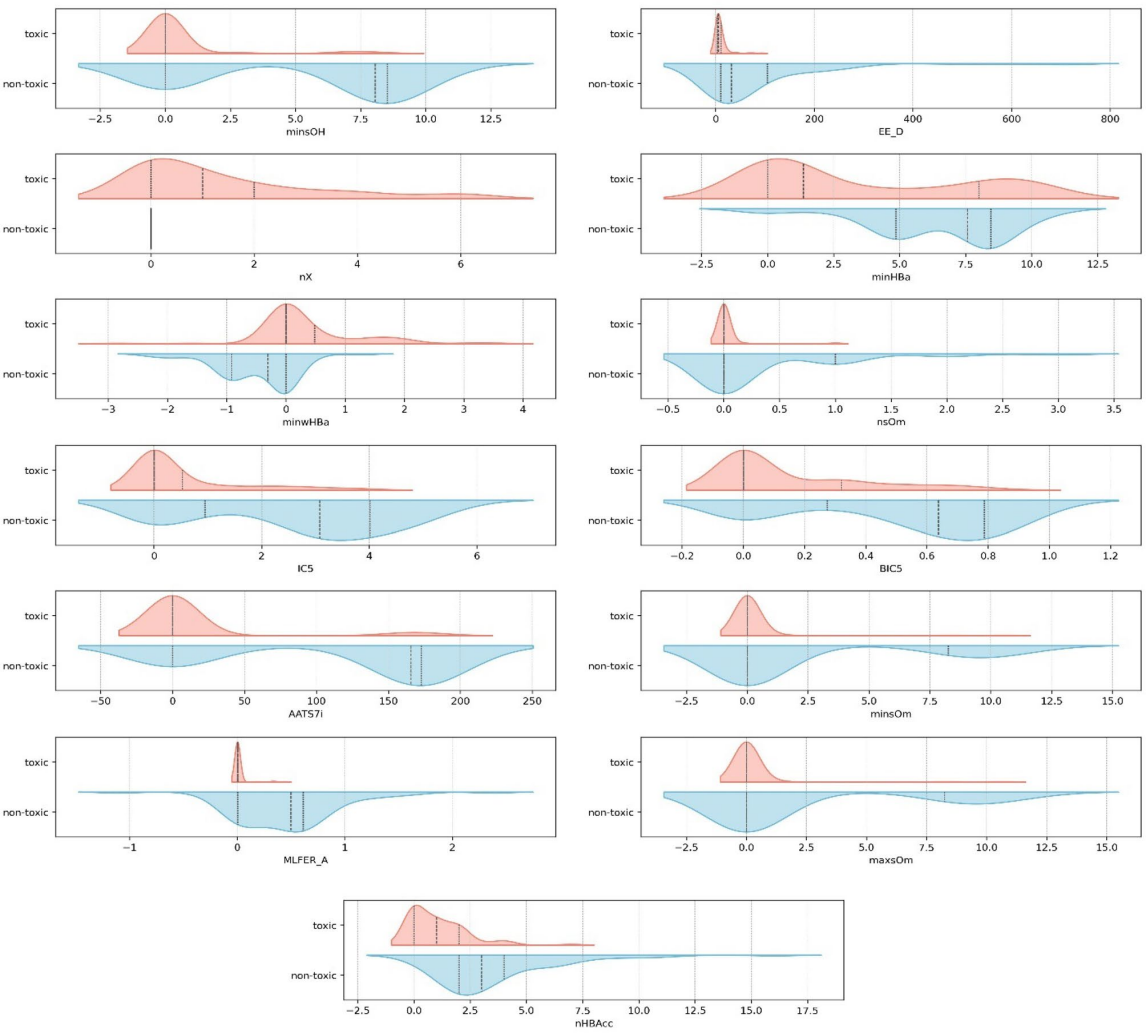
Density distribution of the values of the selected descriptors across toxicity classes. The analytical figures were produced using Python (version 3.12.7) scripts executed in the Spyder integrated development environment under the Anaconda distribution. The official software sources are Python (https://www.python.org/), Anaconda (https://www.anaconda.com/), and Spyder (https://www.spyder-ide.org/).

As a result of this procedure, 13 molecular descriptors satisfying the predefined fold-consistency criterion were retained as the consensus feature set (Table 2). The currently available model included descriptors from the families of the ETA (minsOH, minwHBa, minHBa, nsOm, minsOm, and maxsOm), Atom Count (nX), Information Content (IC5 and BIC5), Autocorrelation (AATS7i), MLFER (MLFER_A), H-Bond Acceptor Count (nHBAcc), and Topological Distance Matrix (EE_D) (Table 3). The distribution of the numerical values of the 13 meta descriptors across the categories (toxic and non-toxic) is shown in Fig. 1.

**Table 2:** Fold-specific molecular descriptors highlighting selection stability across nested cross-validation.

No	Fold 1	Fold 2	Fold 3	Fold 4	Fold 5	Consensus*	Count
1	minwHBa	minwHBa	maxHCsats	minHBa	nsOm	MLFER_A	4
2	nX	minsOm	IC5	minwHBa	AATS7i	minHBa	5
3	nHBAcc	MLFER_A	MLFER_A	MLFER_A	MLFER_A	minwHBa	4
4	minsOH	EE_D	minHBa	nX	minHBa	nX	4
5	minsOm	minHBa	minsOH	nHBAcc	minwHBa	minsOH	4
6	MLFER_A	nX	BIC5	minsOm	nX	AATS7i	3
7	minHBa	minsOH	AATS7i	AATS7i	IC5	minsOm	3
8	maxsOH	AATS7i		EE_D	minsOH	EE_D	3
9	ATS3s	nO		maxsOm	BIC5	nHBAcc	2
10	maxsOm	nsOm		BCUTw-1 h	SsOm	maxsOm	2
11					EE_D	nsOm	2
12						IC5	2
13						BIC5	2

*Within each outer fold, molecular descriptors were selected using the nested cross-validation–based consensus feature selection strategy. The consensus column reports meta-descriptors that were selected in at least two of the five outer folds, representing the most stable features across folds. The count column indicates the number of outer folds in which each descriptor was selected.

**Table 3:** The selected descriptors for our high-performing model.

Identifier	Definition	Class
minsOH	Minimum atom-type E-State: -OH	ETA
nX	Number of halogen atoms (F, Cl, Br, I, At, Uus)	Atom Count
minwHBa	Minimum E-States for weak Hydrogen Bond acceptors	ETA
IC5	Information content index (neighborhood symmetry of 5-order)	Information Content
AATS7i	Average Broto-Moreau autocorrelation - lag 7 / weighted by first ionization potential	Autocorrelation
MLFER_A	Overall or summation solute hydrogen bond acidity	MLFER
nHBAcc	Number of hydrogen bond acceptors	H-Bond Acceptor Count
EE_D	Estrada-like index from topological distance matrix	Topological Distance Matrix
minHBa	Minimum E-States for (strong) Hydrogen Bond acceptors	ETA
nsOm	Count of atom-type E-State: -O-	ETA
BIC5	Bond information content index (neighborhood symmetry of 5-order)	Information Content
minsOm	Minimum atom-type E-State: -O-	ETA
maxsOm	Maximum atom-type E-State: -O-	ETA

ETA: Electrotopological State Atom.

#### Model performance

To construct a structure-aware external validation set, agglomerative hierarchical clustering with Ward linkage was applied to the scaled descriptor space. This approach groups chemically similar observations into the same clusters by iteratively minimizing within-cluster variance. Rather than relying on random sampling, cluster-based splitting was adopted to preserve structural coherence within the development set while assigning entire clusters to the external set^13^. As a result, the development and external sets represent distinct regions of the chemical descriptor space, thereby reducing redundancy between sets and enabling a more stringent assessment of model generalization.

After external set separation, the remaining data were defined as the development set and analyzed exclusively using nested cross-validation. Within each outer cross-validation iteration, the development data were further divided into training and internal validation folds. Consensus feature selection and hyperparameter tuning were jointly performed using only the training folds of the inner cross-validation loop. The most stable meta-descriptor set and hyperparameter configuration identified across iterations were then fixed. Using the fixed meta-descriptor set, the final model was re-trained on the full development set, with nested cross-validation employed solely for hyperparameter tuning, and subsequently evaluated once on the held-out external set. Nested cross-validation on the development set showed stable performance across folds (Table 4). All models achieved high outer-fold discrimination (AUC ≥ 0.974), with minimal differences between inner- and outer-fold estimates. Performance variability across folds remained limited. This final step was applied consistently to the RF, XGBoost, and CatBoost models. External performance metrics are in the Table 4.

**Table 4:** Nested cross-validation results (5-fold outer cross validation) of the developed models on the development dataset.

			Outer mean±S.D.			Inner Best^*^ mean±S.D.
	AUC	ACC	Balanced ACC	F1-Score	MCC	AUC
SVM	0.980±0.015	0.901±0.040	0.902±0.039	0.901±0.040	0.803±0.079	0.987±0.005
RF	0.980±0.010	0.883±0.044	0.884±0.043	0.883±0.044	0.773±0.088	0.982±0.008
XgBoost	0.977±0.008	0.920±0.017	0.920±0.017	0.920±0.017	0.842±0.035	0.978±0.006
CatBoost	0.974±0.022	0.920±0.041	0.921±0.041	0.920±0.041	0.844±0.083	0.980±0.008

SVM: Support Vector Machine; RF: Random Forest; XGBoost: Extreme Gradient Boosting; CatBoost: Categorical Boosting; AUC: Area Under the Curve; ACC: Accuracy; MCC: Matthews Correlation Coefficient; S.D.: Standard Deviation.

* Inner best AUC corresponds to the mean 3-fold cross-validation AUC of the optimal hyperparameter configuration selected within each outer fold.

**Table 5:** Predictive performance evaluated on the external set.

SVM	ROC-AUC	Accuracy	Balanced Accuracy	Specificity	Sensitivity	MCC	F1-Score	Kappa
	0.9704	0.8571	0.8635	0.7895	0.9375	0.7270	0.8571	0.7163
RF	0.9243	**0.8571**	0.8585	0.8421	0.8750	0.7147	0.8566	0.7135
XgBoost	0.9572	**0.8571**	0.8585	0.8421	0.8750	0.7147	0.8566	0.7135
CatBoost	0.9605	**0.8571**	0.8571	0.8421	**0.9375**	0.7147	0.8566	0.7135

SVM: Support Vector Machine; RF: Random Forest; XGBoost: Extreme Gradient Boosting; CatBoost: Categorical Boosting; ROC-AUC = Area Under the Receiver Operating Characteristic Curve; MCC: Matthews Correlation Coefficient.

As shown in Table 5, the models exhibit comparable overall accuracy; however, pronounced differences emerge when error-type sensitive metrics are considered. Given that false negatives, misclassifying toxic compounds as non-toxic, represent the most critical error mode in this context, model performance was primarily evaluated using sensitivity (recall for the toxic class), balanced accuracy, and Matthews correlation coefficient (MCC). The SVM model achieved the highest sensitivity (0.9375, shared with CatBoost) while simultaneously maintaining the strongest overall discrimination (ROC-AUC = 0.9704) and the highest MCC (0.7270), indicating a favorable balance between false negative control and overall classification reliability. Although tree-based models demonstrated higher specificity, their comparatively lower MCC and ROC-AUC values suggest a less optimal trade-off between error types. Collectively, these results support the selection of SVM as the primary model for downstream analysis and external validation, particularly under a risk-averse setting prioritizing the minimization of false negatives.

The SVM model was tuned exclusively on the training data using a nested cross-validation framework, in which hyperparameter optimization was performed within the inner loop via stratified 5-fold cross-validation and grid-search optimization, while model evaluation was conducted in the outer loop. Neither the inner validation folds nor the external dataset were used at any stage of model selection or hyperparameter tuning, ensuring an unbiased assessment of generalization performance.The optimal SVM configuration was identified with a regularization parameter of $\:C$ = 128 and and a radial basis function kernel parameter $\:\gamma\:$= 0.1. The development dataset exhibited balanced class distributions between the toxic (class 1 = 82) and non-toxic (class 0 = 80) classes. When evaluated on the external test set, the optimized SVM model yielded the confusion matrix $$\:\left[\begin{array}{cc}15&\:4\\\:1&\:15\end{array}\right]$$ corresponding to one false negative and four false positives. Given that false negatives represent the most critical error mode in this study, the low false negative count highlights the suitability of the SVM model for risk-averse toxicity screening.

All predictive models were subjected to a label-randomized permutation test (*n* = 10.000), yielding p-values < 0.0001. These results indicate that the discriminative performance of the models on the external validation set is highly statistically significant and unlikely to arise from random label–score associations.

#### Model explanation

**Fig. 2:**
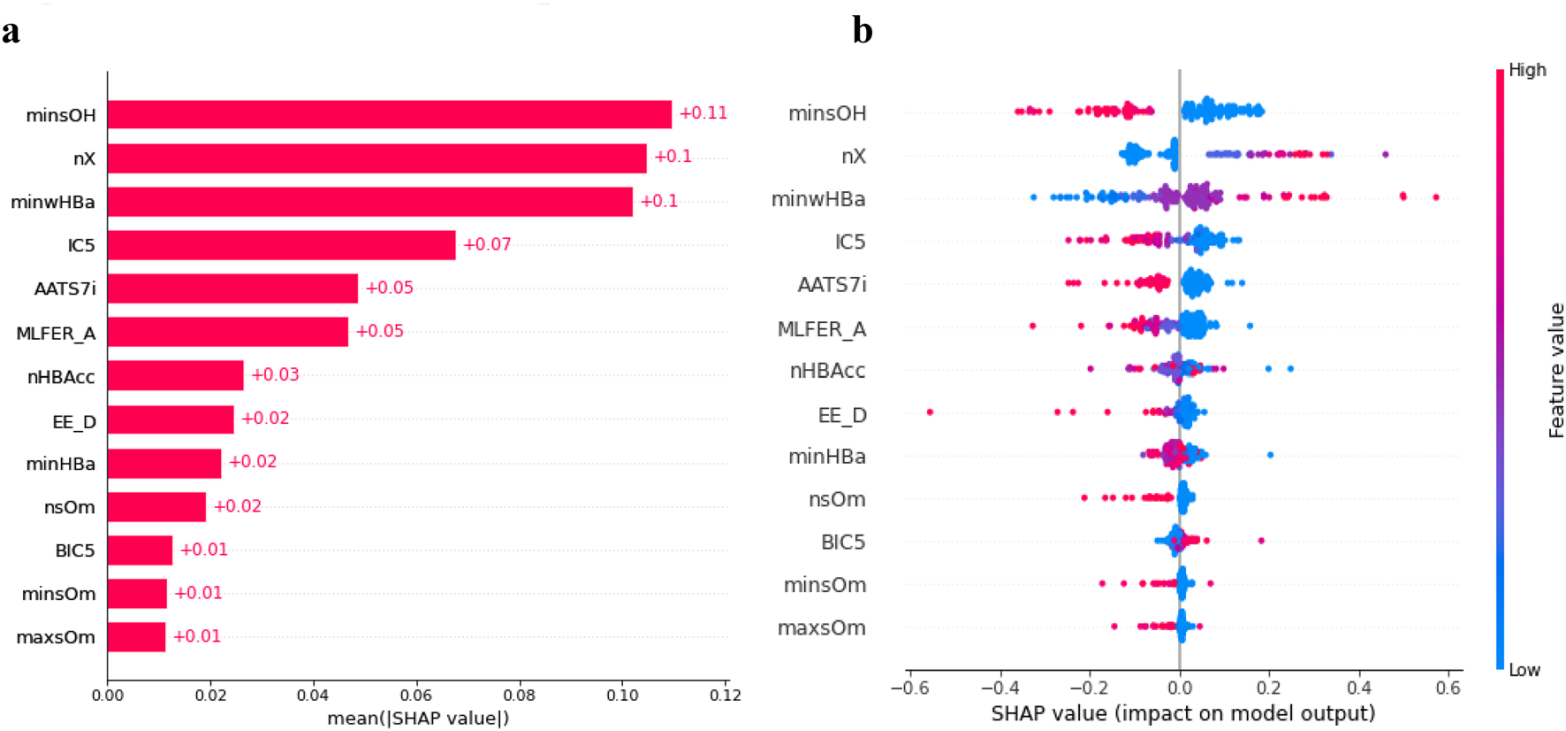
SHAP Global feature explanation. **a**, Bar plot. **b**, Beeswarm plot. The analytical figures were produced using Python (version 3.12.7) scripts executed in the Spyder integrated development environment under the Anaconda distribution. The official software sources are Python (https://www.python.org/), Anaconda (https://www.anaconda.com/), and Spyder (https://www.spyder-ide.org/).

**Fig. 3:**
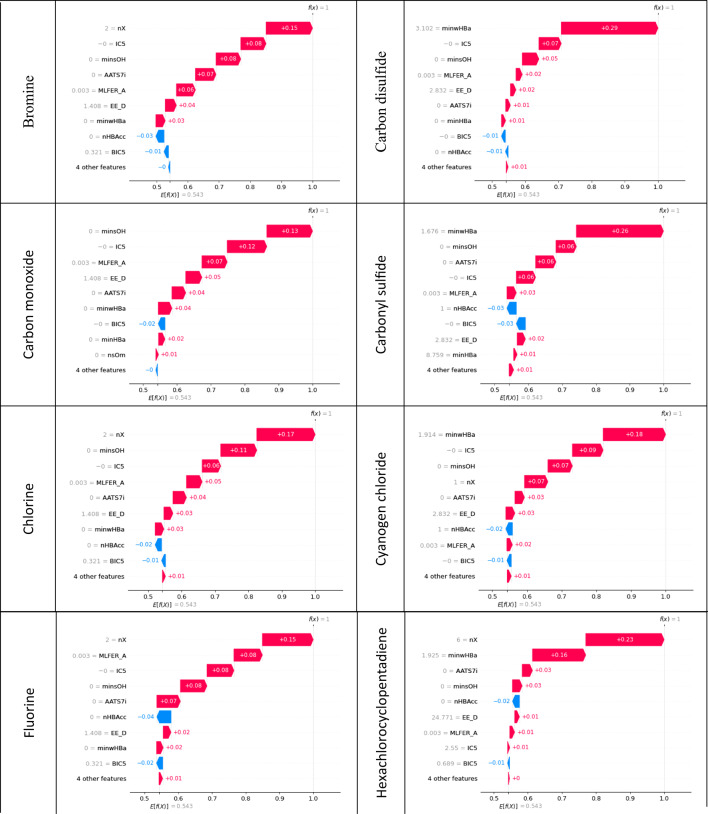

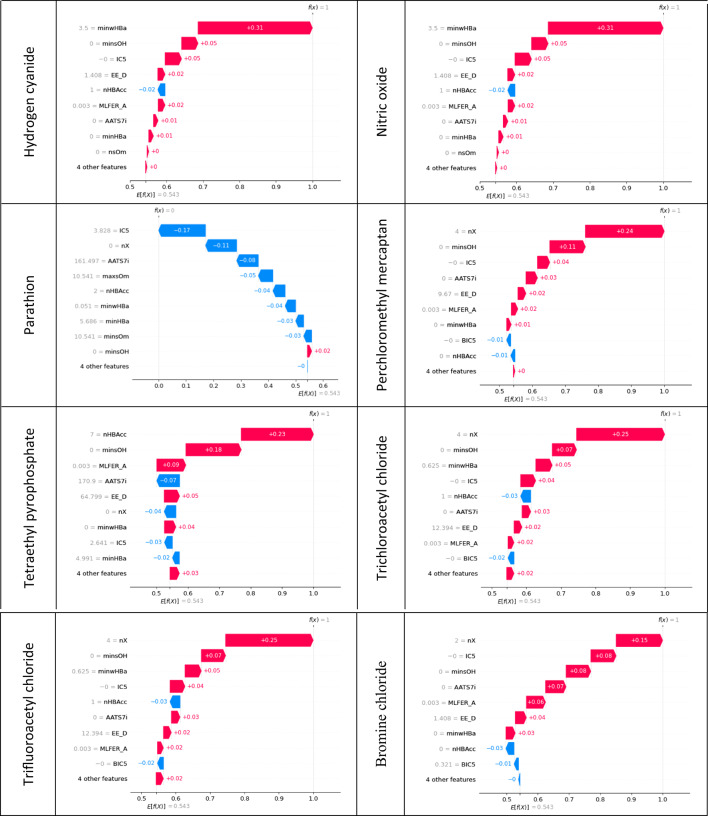
Local explanation of specific descriptors related to the acute inhalation toxicity for toxic industrial chemicals (TICs) in the test set using SHAP waterfall plots. The analytical figures were produced using Python (version 3.12.7) scripts executed in the Spyder integrated development environment under the Anaconda distribution. The official software sources are Python (https://www.python.org/), Anaconda (https://www.anaconda.com/), and Spyder (https://www.spyder-ide.org/).

Following model selection based on predefined external validation criteria (prioritizing sensitivity, followed by MCC and ROC-AUC), the SVM model was selected as the final predictive model. SHAP-based explainability analysis was subsequently conducted exclusively on this model^14^. Global explanations describing the relationship between molecular descriptors and toxicity risk are presented in Fig. 2, followed by local explanations for individual chemicals. Local explanations for all test set compounds are provided in Fig. 3.

The SHAP summary graph (Fig. 2a) presents the SHAP values of various molecular descriptors predicted using the SVM model. The horizontal axis represents the SHAP values and indicates the impact of each descriptor on the model output. The vertical axis shows the features of all chemical components according to their cumulative SHAP values. The five most influential features were minsOH, nX, minwHBa, IC5, and AATS7i, listed in order of contribution, while EE_D, minHBa, nSOm, BIC5, minsOM, and maxOm descriptors had relatively limited impact.

Each data point on the graph represents a chemical, with a color gradient from red (high values) to blue (low values) indicating the relative magnitude of property values. Data points to the right of the vertical line correspond to a higher probability of a compound being classified as toxic (positive), whereas negative SHAP values on the left indicate a higher likelihood of classification as non-toxic.

Figure 2b shows minsOH as the most influential among the top 13 descriptors. Lower minsOH values are correlated with an increased probability of toxicity. A similar trend is observed for IC5, AATS7i, MLFER_A, nHBAcc, EE_D, minHBa, nsOM, minsOM, and maxOm. Conversely, nX, minwHBa, and BIC5 values correspond to a greater likelihood of toxicity.

In the examination of the local SHAP graphs for the 16 toxic molecules in the test set, the most significant descriptors were nX (8 out of 16), minwHBa (5 out of 16), nHBAcc (1 out of 16), minsOH (1 out of 16), and IC5 (1 out of 16). nX ranked top as the most frequently recurring descriptor contributing positively to the toxic effect (for Bromine chloride, Bromine, Chlorine, Fluorine, Hexachlorocyclopentadiene, Perchloromethyl mercaptan, Trichloroacetyl chloride, and Trifluoroacetyl chloride). minwHBa ranked second (for Carbon disulfide, Carbonyl sulfide, Cyanogen chloride, Hydrogen cyanide, and Nitric oxide). Descriptors showing a positive correlation with toxicity have been identified: nHBAcc (for Tetraethyl pyrophosphate), and minsOH (for Carbon monoxide). Finally, IC5 showed a negative correlation with toxicity (for Parathion).

#### Pilot compound selection

The goal of this step is to identify the highest-risk chemical based on risk prioritization. High-risk chemicals for inhalation toxicity were evaluated for their suitability for descriptor modification. Substances classified as “High” under the Hazard Index (HI) code received priority. Then, among compounds in the “High” category, we focused on chemicals coded H330 (*n* = 13), which pose the highest risk for acute inhalation toxicity according to the Globally Harmonized System of Classification and Labeling of Chemicals (GHS) hazard codes^15^. Chemicals with detailed respiratory toxicity parameters were accessed (*n* = 5). The five candidate compounds were assessed based on respiratory toxicity parameters for different exposure durations, as shown in Table 6. These parameters were used as the criteria for the Technique for Order Preference by Similarity to Ideal Solution (TOPSIS) method, which was implemented in the Python environment using the *pyMCDM* library^16^. The detailed mathematical steps are provided in Supplementary Eq. 1, while the ranking results are presented in Table 6. According to these results, Arsine was identified as the pilot chemical with the highest-risk profile, initiating the subsequent optimization of targeted descriptors. Following Arsine, the subsequent priorities for modification included Phosgene, Fluorine, Formaldehyde, and Hydrogen fluoride.

**Table 6:** Criteria for pilot compound selection and corresponding TOPSIS results.

	Evaluation Criteria					TOPSIS Results	
	IDLH (ppm)	ERPG-3 (ppm)	RfCa (mg/m^3^)	REL-TWA (ppm)	RfC (mg/m^3^)	Score (Q_i)_	Rank
Arsine	3	1.5	2.00E-04	0.005	5.00E-05	0.9841	1
Phosgene	2	1.5	4.00E-03	0.1	3.00E-04	0.9621	2
Fluorine	25	20	1.55E-02	0.1	1.30E-02	0.5033	3
Formaldehyde	20	40	4.91E-02	0.016	7.00E-03	0.4399	4
Hydrogen fluoride	30	50	1.64E-02	3	1.40E-02	0.2480	5

IDLH: Immediately Dangerous to Life and Health; ERPG-3: Life-threatening effects after 1-hour inhalation exposure; RfCa: Inhalation Acute Reference Concentration; REL-TWA: Average exposure limit over 8 h/day, 40 h/week; RfC: Inhalation Chronic Reference Concentration^17–20^. In the TOPSIS analysis, all criteria were considered cost-type (minimize) objectives.

#### SHAP analysis for the pilot compound

**Fig. 4:**
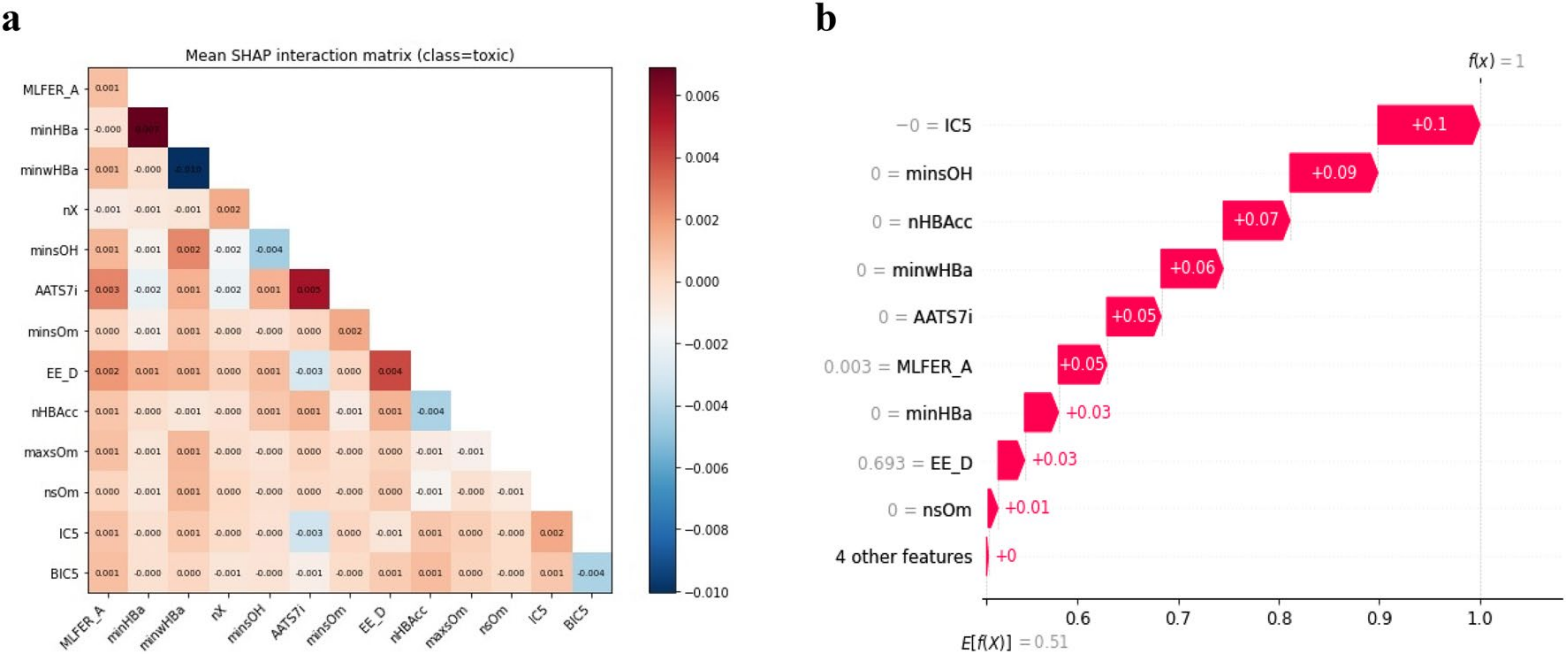
SHapley Additive exPlanations (SHAP) for Arsine. **a**, SHAP Interaction. **b**, Local explanation. The analytical figures were produced using Python (version 3.12.7) scripts executed in the Spyder integrated development environment under the Anaconda distribution. The official software sources are Python (https://www.python.org/), Anaconda (https://www.anaconda.com/), and Spyder (https://www.spyder-ide.org/).

Before initiating descriptor optimization for the pilot compound (Arsine), potential feature dependencies were examined using a SHAP interaction heatmap. For consistency in reporting, the interval − 0.3 to + 0.3, commonly cited as a weak range for Pearson’s coefficient^21^, was adopted solely as a reference threshold. As indicated by the SHAP interaction patterns in Fig. 4a, the selected descriptors do not exhibit strong mutual dependencies, supporting their use in subsequent descriptor perturbation and optimization analyses.

Based on the local explanation of the pilot compound, the SHAP waterfall plot demonstrates the contribution of individual descriptors to the model prediction. These results highlight the dominant role of specific molecular descriptors in shaping the model outcome. According to Fig. 4b, molecular descriptors such as IC5, minsOH, nHBAcc, minwHBa, AATS7i, MLFER_A, minHBa, EE_D, and nsOm provided strong positive effects, directing the prediction from the average value (E[f(x)] = 0.51) towards the predicted value (f(x) = 1).

**Fig. 5:**
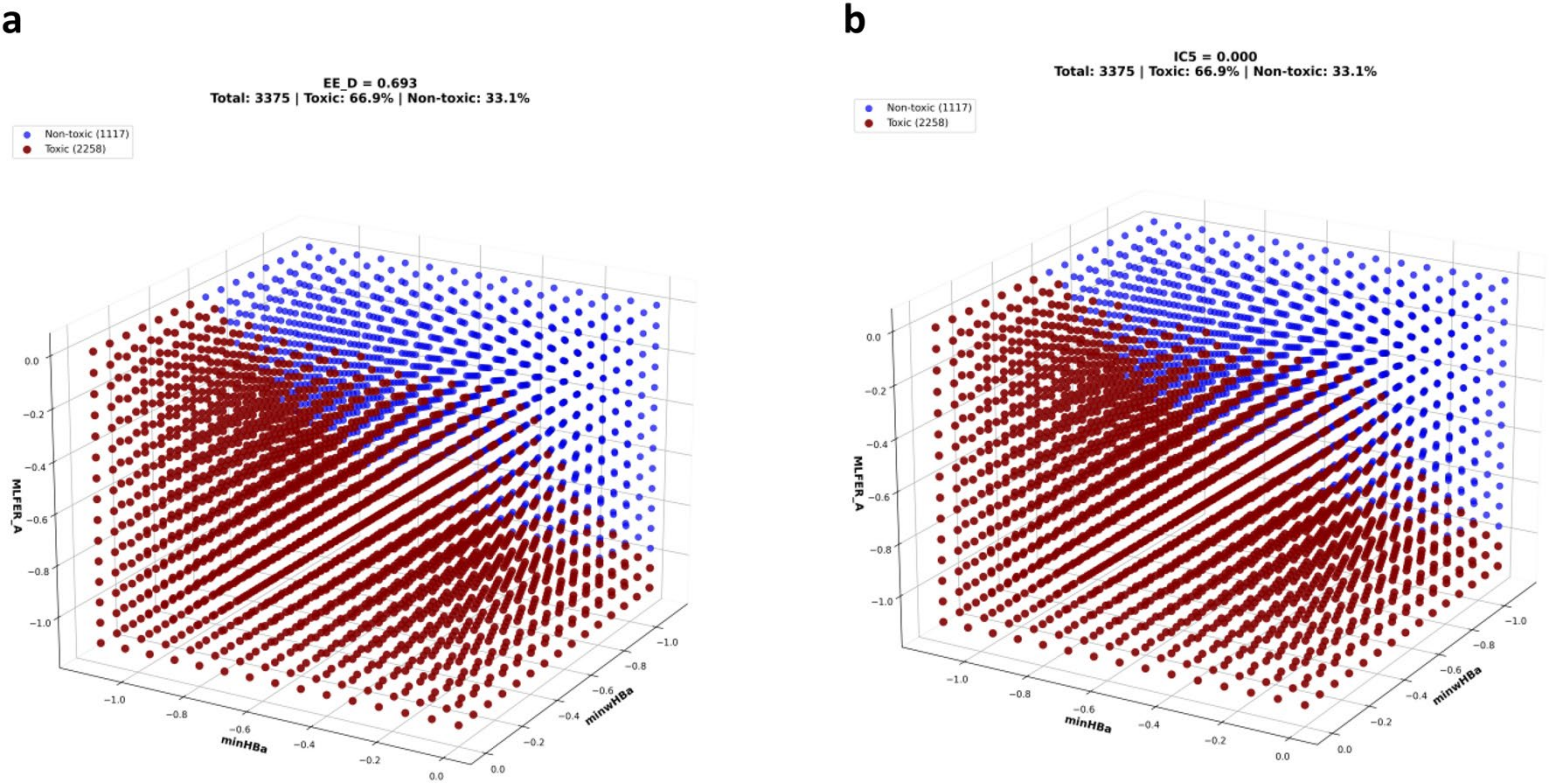
Decision boundary plot for virtual molecular representations. **a**, Compounds at fixed EE_D value. **b**, Compounds at fixed IC5 value. The analytical figures were produced using Python (version 3.12.7) scripts executed in the Spyder integrated development environment under the Anaconda distribution. The official software sources are Python (https://www.python.org/), Anaconda (https://www.anaconda.com/), and Spyder (https://www.spyder-ide.org/).

These findings indicate that key descriptors drive toxicity prediction and require independent consideration. A grid design was created for Arsine, and to avoid complex models, three key descriptors (minwHBa, minHBa, and MLFER_A) were reduced by 0.08 units over 15 iterations based on SHAP contribution values as a reference. As a result of this process, 3,375 virtual molecular representations in descriptor space were generated and re-evaluated using the primary prediction model (SVM with $\:C$ = 128, $\:\gamma\:$= 0.1.), of which 1,117 compounds were classified as non-toxic. For virtual molecular predicted as non-toxic, the mean predicted toxicity probability was 0.187 (95% CI: 0.179–0.195), whereas virtual molecular predicted as toxic exhibited a substantially higher mean toxicity probability of 0.853 (95% CI: 0.847–0.859). The model performs toxic/non-toxic classification using the minwHBa value (-0.52 threshold), the MLFER_A value (-0.59 threshold), followed by the minwHBa (-0.84 threshold). The decision boundaries are more clearly visible in the 3D graphs presented in Fig. 5. Depending on the research objective, the analysis can also be conducted using other descriptor groups. In addition, decision-tree analyses were applied to examine the effects of the descriptors contributing most to toxicity and to define their corresponding reference ranges.

**Fig. 6:**
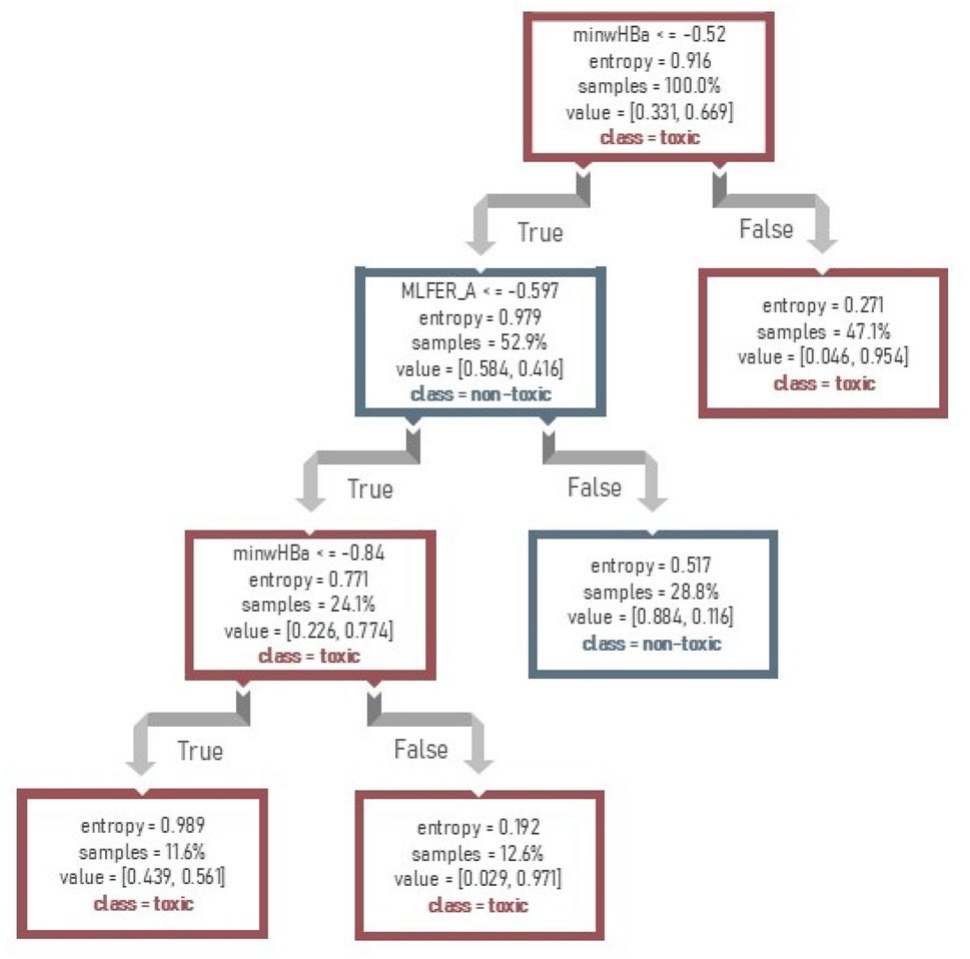
Hierarchical decision tree representation for toxicity prediction of virtual molecular representations. The analytical figures were produced using Python (version 3.12.7) scripts executed in the Spyder integrated development environment under the Anaconda distribution. The official software sources are Python (https://www.python.org/), Anaconda (https://www.anaconda.com/), and Spyder (https://www.spyder-ide.org/).Created by the authors using Microsoft PowerPoint 2019 MSO (Version 2508, https://www.microsoft.com).

The decision tree presented in Fig. 6 demonstrates the hierarchical decision structure of molecular descriptors in toxicity prediction of virtual molecular representations.

To further formalize these threshold patterns, a decision tree was trained on the SVM-generated class assignments of the virtual compounds to approximate the underlying decision structure. Tree complexity was optimized via grid search (max_depth ∈ {3, 5, 10}, max_leaf_nodes ∈ {2, 4}, min_samples_leaf ∈ {2, 4, 6, 10}, criterion ∈ {gini, entropy, log_loss}). The optimal configuration (criterion = entropy, max_depth = 3, max_leaf_nodes = 4, min_samples_leaf = 2) achieved an agreement accuracy of 0.921 with the SVM predictions, confirming that the extracted tree structure reliably captures the dominant decision thresholds identified in the 3D analysis.

Based on the decision-tree analysis, virtual molecular representations are predominantly identified as non-toxic when the hydrogen-bond acceptor descriptor (minwHBa) exceeds − 0.52 and the MLFER_A parameter remains below − 0.597. In contrast, lower minwHBa values are consistently associated with toxic classifications, regardless of downstream descriptor splits. These model-derived decision thresholds provide an interpretable rule set that supports rational screening of virtual compounds and facilitates the prioritization of candidates for subsequent experimental validation.

## Discussion

Green toxicology aims to design safer substances by minimizing or preventing potential toxicity at an early stage, thereby reducing the need for post-development testing^5^. The introduced DETOX-QSAR model was developed to advance the green toxicology paradigm. Unlike typical QSAR models, DETOX-QSAR focuses on redesigning molecular structures for achieving specific outcomes. Our model claims to eliminate adverse effects by modifying molecular descriptors. Briefly, we aimed to prevent toxicity at the molecular level by optimizing targeted descriptors. Firstly, the local SHAP graphs revealed distinctive descriptors contributing to the target effect at a molecular level. Then, using the Grid Search, we guided a toxic substance toward the non-toxic domain by manipulating its descriptors provided by local SHAP. While we mitigated multicollinearity through consensus feature selection, the interpretation of SHAP values for individual descriptors should be considered in the context of potential feature interactions. In R&D, this mechanistic information can facilitate developing safer alternatives in chemical synthesis process. The most notable aspect of our research, a new insight not previously shown in existing studies, is the identification of key descriptors at the molecular level using XAI techniques and the complete elimination of toxic effects without relying on laboratory experiments.

The descriptors were evaluated as explainable signals that the QSAR model associates with toxicity. The controlled perturbations applied to these signals indicate a shift of the molecules toward the non-toxic class in the model output; this transition does not represent a chemically synthesizable transformation, but rather a model-based class change. This approach provides a framework for model-based optimization.

In local SHAP graphs, the top three most frequently recurring descriptors are nX and minwHBa. These descriptors have contributed positively to the toxic effect. nX is related to halogen substitution and is commonly associated with persistence, bioaccumulation, and toxicity behaviors of chemicals^22^. Halogens pose risks to lungs, causing inhalation toxicity that results in hypoxemia, dyspnea, pneumonitis, airway obstruction, acute respiratory distress syndrome, and pulmonary edema^23^. In local SHAP explanations of TICs, the most frequently highlighted descriptor following nX is minwHBa. It represents minimum e-states for weak hydrogen bond acceptors (HBA)^24,25^. minwHBa’s positive contribution to toxicity shows that even the weakest receptor can play a role in biological interactions. We suggest that removing functional groups classified as weak HBA (especially halogens such as F, Cl, and Br) from the molecular structure could mitigate toxic effects^26^. Supporting our findings, Janicka and Śliwińska (2022) reported that an increase in the number of HBA decreases the lethal dose 50 (LD₅₀) values, thereby increasing toxicity^27^.

In this study, meaningful descriptors (for interpreting the changes) for the selected pilot molecule (Arsine) were identified as minwHBa, minHBa, and MLFER_A, and toxicity prediction was directed toward the non-toxic area through in silico modifications. Both minwHBa and minHBa are ETA-based descriptors associated with HBA characteristics. HBDs and HBAs are mentioned as two critical issues in drug design^28^. In a study by Felgenträger et al. (2013), increasing the number of HBAs in the drug molecule enhanced bactericidal activity against *S. aureus* and *E. Coli*^29^. Consistent with the literature, our study found that an increase in HBAs is associated with toxic effects. For MLFER_A, given that Arsine lacks typical HBD groups, the reduction of MLFER_A should focus on minimizing interactions-based electronic and polarization effects. Restricting the molecule to a bound or immobilized state can limit its ability to interact freely, thereby reducing the acidic or reactive character represented by MLFER_A.

The critical molecular descriptors identified in this study can facilitate guidance toward the non-toxic region, although their use is not mandatory. A molecule may possess numerous physicochemical properties that could induce toxic effects. This study identified the descriptors most strongly correlated with toxicity and demonstrated their interpretive significance in explaining the model’s predictions. The proposed approach provides a general framework for reducing toxic effects by modifying descriptors. During the chemical design process, various or more appropriate descriptors can be optimized using the same principles, depending on the study’s specific goals and application conditions.

Molecular descriptors may not directly represent a single structure, but rather reflect various physicochemical properties that can correspond to multiple molecular structures. Direct one-to-one mapping between descriptors and specific functional groups is non-trivial. Therefore, in practice, changing a targeted identifier can be achieved through various strategies based on different molecular structure modifications. These strategies should be selected considering factors such as laboratory conditions and the modified molecule’s synthesizability, activity, and stability. A main limitation of our study is that it does not directly address the shift from the modified descriptor set to an applicable chemical structure. The primary reason for this is the variability in TICs’ uses and the need to prioritize preserving targeted activity in molecular design. The wide range of applications of the compounds makes proposing a single structural change challenging. However, in application scenarios with well-defined targets, in silico - driven molecular design may help reduce toxicity while maintaining biological activity. In this context, this study’s outputs could guide molecular modifications applicable in real-world scenarios in future research. Specifically, a potential future direction would be to incorporate the optimized descriptor values as constraints into *de novo* molecular generation algorithms, including Variational Autoencoders and transformer-based models.

Our model provides a fast, affordable, and ethical approach for predicting the sudden and lethal inhalation risk caused by chemicals. This allows manufacturers to accelerate preliminary toxicity testing before launching new chemicals. Furthermore, the computational method simplifies regulatory compliance by identifying the toxicity profiles of industrial chemicals. It also assists emergency response teams in quickly evaluating the potential hazards of unknown compounds during industrial accidents, thereby minimizing environmental and human risk.

Predictive models have supported international efforts to control the use of TICs/CWAs^30^. Since TICs are overshadowed by CWAs in terms of toxicity severity, current QSAR models mainly focus on CWA toxicological traits, biodegradation, and reactivity profiles^30–32^. Moreover, computational modeling studies on TICs are limited, and without a classification-based QSAR model for TICs like ours, direct comparisons between models remain challenging. In the literature, QSAR models for toxic chemicals are generally based on regression and mostly focus on a specific endpoint, such as lowest-observed-adverse-effect level (LOAEL), median LD_50_, half maximal inhibitory concentration (IC_50_), and no-observed-adverse-effect level (NOAEL) based on acute toxicity by oral exposure^33–36^. Although oral toxicity studies are valuable, the primary risk associated with TICs is the emission of massive amounts of liquefied, pressurized gases. Since most TICs are released as vapors, inhalation is usually considered the main route of exposure for assessing their toxicity^37^. Unlike previous studies, we used acute inhalation toxicity data to assess TICs. On the other hand, some modeling studies using different bacterial strains/cell lines as test systems specifically focused on industrial chemicals such as aromatic brominated, chlorinated disinfection byproducts^38^, nitro-substituted benzenes^39^, dimethyl formamide, methyl ethyl ketone, and toluene mixtures^40^. In contrast, our comprehensive dataset encompassed various industrial chemical groups, with toxicity markers derived from data collected in humans, mice, and rats. Since there are no specific QSAR models for TICs in the literature, the model comparison was conducted using an acute inhalation toxicity classification models applicable to general chemical groups. In the Fuadah et al. (2024) study, the consensus model combining RF, XGBoost, and SVM algorithms achieved an approximately 0.73 success rate with sensitivity 0.78 and specificity 0.69^41^. Chushak et al. (2023) reported a 0.75 success rate with a Message Passing Neural Network-based model^42^. Borba et al. (2022) had a 0.70 success rate with RF^43^, while our RF model reached a success rate of 0.89. Regression-based QSAR models for predicting rodent inhalation toxicity LCt₅₀ for different types of industrial chemicals have been widely reported^44–46^. While these approaches focus on predicting the quantitative level of toxicity, the current study presents a classification-based framework for assessing the presence of acute inhalation toxicity risk.

Our dataset is medium-to-small scale, as the reported TICs’ list is limited. This situation prevents us from creating an additional external dataset and testing the model on an external validation set. In the future, defining new TICs will allow for more comprehensive and reliable validation of the model using external datasets. It will also enable the assessment and recalibration of the model’s performance across different chemical spaces, thereby improving its generalizability and predictive power. Although the dataset is accessible and the methodological steps are clearly presented, reproducing the model remains difficult and requires significant domain-specific knowledge. Despite the several advantages of the model, further research is necessary to reach a final decision, as with other toxicology studies. Maintaining consistently high safety standards is crucial in preventing unexpected damage.

In conclusion, the DETOX-QSAR is the first model to evaluate the acute inhalation risk of TICs and assist in hazard elimination at the molecular level. The study introduces the novel QSAR-driven descriptor optimization strategy aimed at guiding the redesign process to eliminate toxicity. Our study transitions from predictive modeling to mechanistic insight. The industrial safety model can ensure regulatory compliance, support proactive risk assessment, quickly detect toxicities in emergencies, provide early warnings, and reduce occupational exposure. Beyond predicting the toxicity risk of TICs, the model has provided data on the chemical manufacturing phase, particularly identifying the necessary alterations to the molecular variants of each compound. This study shows that early in silico identification of key molecular descriptors can help proactively assess potential toxicity. Our model can contribute to the production of green chemicals and the sustainable management of chemicals. In this approach, we aim to safeguard both the environment and human health by encouraging the production of green chemicals.

## Methods

Recent advances in data resources and computational algorithms have placed AI at the forefront of current toxicological research. ML has improved risk assessment processes in industrial toxicology by providing legal convenience alternatives to traditional methods. Applications range from predicting toxic compounds to improving contaminant removal processes and developing high-content screening techniques^47^.

The proposed approach in this study involves predicting TICs using ML algorithms and presenting model explanations to the end user regarding the degrees and directions of the toxic effects caused by the molecular descriptors considered as explanatory variables of the model. This study presents a QSAR-based prediction model using ML algorithms, followed by molecular-level toxicity reduction optimization called DETOX. The DETOX-QSAR model is introduced to the literature.

**Fig. 7:**
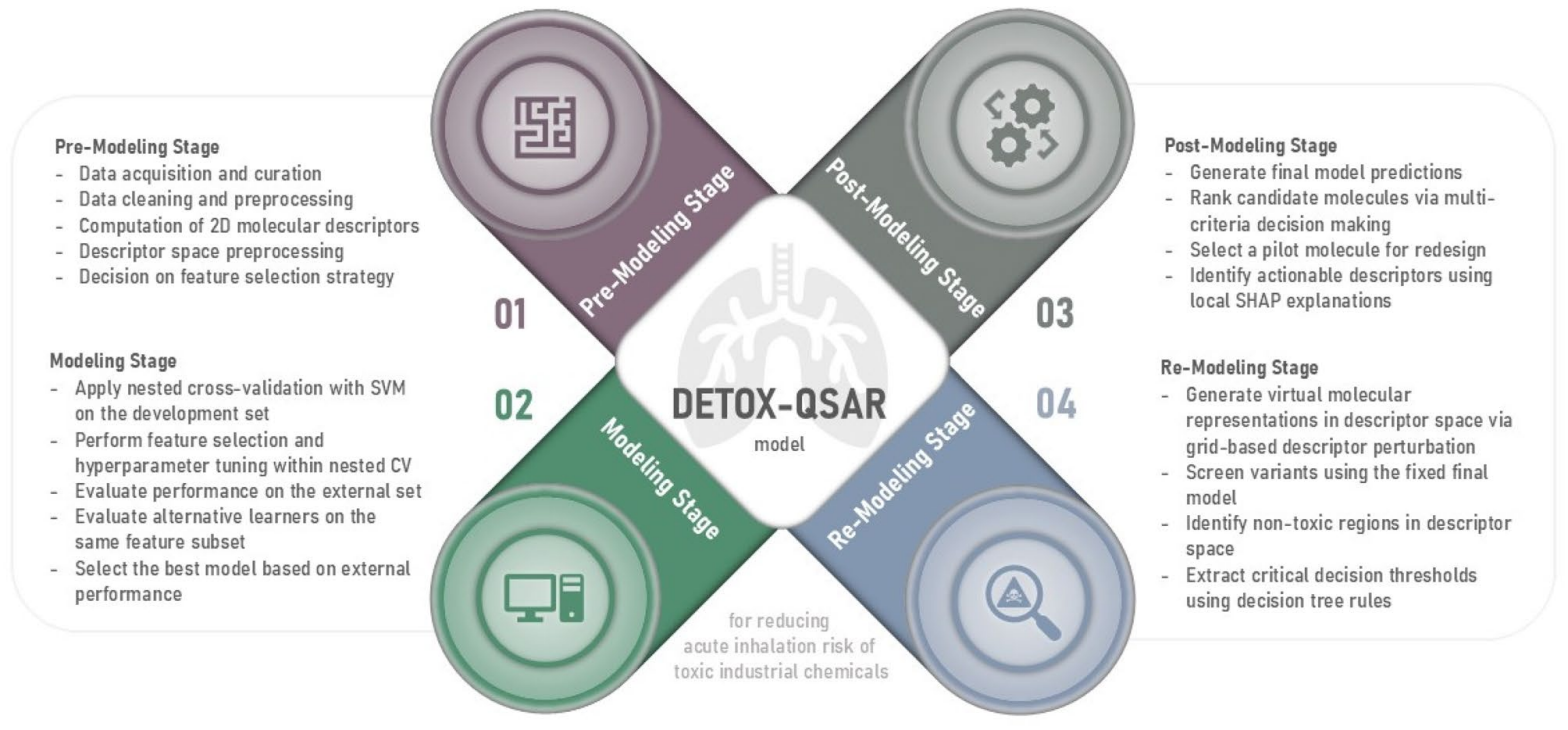
The DETOX-QSAR framework. Created by the authors using Microsoft PowerPoint 2019 MSO (Version 2508, https://www.microsoft.com).

Building the model involves several essential steps, from initial data preparation to detailed statistical analysis. Our research framework is visualized in Fig. 7. To obtain a strong and reliable prediction model, it is essential to identify certain molecular descriptors that may lead to a simpler or highly complex model - potentially causing underfitting or overfitting - approaching the target function. In this context, a feature engineering process has been proposed where a consensus approach with well-known feature selection methods is preferred to reveal the optimal feature set. The SVM method, which is based on distance calculation, and ensemble learning algorithms such as RF, XGBoost, and the recently introduced CatBoost were used to develop the prediction model. The Grid Search method was used to optimize the internal parameters of the algorithms that strongly impact the model prediction. To prevent bias arising from feature selection and model optimization, feature selection was embedded within a nested cross-validation framework, while an independently constructed external dataset was used exclusively for final model evaluation. Finally, the best prediction model identified was explained using SHAP. Critical descriptors influencing the target effect were displayed for each compound using local SHAP annotations. The most risky chemical in the test set was identified through a layered process, and its descriptors were optimized via grid search to enhance its safety profile.

## Data collection

The binary dataset (n_total_=197) included toxic and safe (non-toxic) chemicals for acute inhalation toxicity. The group of toxic chemicals consisted of all available TICs (*n* = 98) from the Occupational Safety and Health Administration (OSHA) website^2^. In this study, only TICs were assessed for acute inhalation toxicity because of their potential for immediate lethal effects. Safe chemicals were collected from “Green Circle” compounds (*n* = 99) listed on the United States Environmental Protection Agency’s (EPA) Safer Chemical Ingredients List (SCIL)^8^. The OSHA TIC list and the EPA Green Circle list were used to define the candidate molecule pool and were not used for labeling. The endpoint in this study was defined according to the GHS acute inhalation toxicity classification based on LCt₅₀ values. The GHS acute inhalation toxicity classification of each compound in the candidate molecule pool was examined individually, and binary (toxic/non-toxic) labeling was performed based on these classifications. The toxic class consisted of compounds assigned to at least one of the GHS hazard codes H330-H335. The non-toxic class consisted of compounds that were not assigned any GHS H330–H335 hazard codes and that were not classified for acute inhalation toxicity based on the available regulatory data. Dataset details are presented in the Supplementary Note 1 (Supplementary Tables S1, S2, and S3).

## Molecular feature processing

### Calculation of the features

2D descriptors are widely used in toxicity prediction because they include features such as atom count, bond type, and topological indices, which are easy to compute and represent the basic molecular structure^48^. In this study, molecular features were sourced from 2D structures available in the PubChem database^20^ and the open-source PaDEL software^10^ was used as a calculation tool. Descriptors calculated for the entire dataset are provided in the Supplementary File 1.

### Feature selection methods

The performance of ML models in prediction largely depends on the quality and homogeneity of the datasets used. Providing high-quality datasets is crucial for reliable model development^49^. Our goal was to identify the key subset-features that create the strongest model and guide molecular-level modifications. Accordingly, we proceeded to the feature selection phase to select critical descriptors.

Our DETOX-QSAR modeling aims to precisely evaluate the relationship between molecular descriptors and target toxicity. In this context, QSAR models can be developed that use a large set of molecular descriptors as input and toxicity direction as output. On the other hand, the high dimensionality of molecular descriptors can lead to overfitting and multicollinearity problems in predictive modeling. Multicollinearity refers to high intercorrelations among descriptors and complicates model interpretation and stability^50,51^. To overcome this problem, ElNet, LASSO, RF, and RF-RFE methods are frequently used^51–54^.

#### Least Absolute Shrinkage and Selection Operator (LASSO)

The LASSO estimator, introduced by Tibshirani (1996), is also frequently used as a feature selection technique in high-dimensional datasets in ML processes^55^. The LASSO estimator is valuable due to its ability to effectively reduce the number of features by applying the L1-norm penalty to some of the coefficients given in Eq. 1^56^.

1 $$\:\sum\:_{i=1}^{M}\:p\left({\beta\:}_{hj};\lambda\:\right):=\lambda\:\sum\:_{i=1}^{M}\:\left|{\beta\:}_{kj}\right|$$

#### Elastic Net Regularization Scheme (ElNet)

Zou and Hastie (2005) proposed ElNet, a method that balances model sparsity and accuracy for selecting related feature groups^57,58^. It can benefit from the important features of LASSO and Ridge regression, with the control parameter α∈[0,1] between the L1 (encouraging some of them to become exactly zero) and L2 (preventing them from growing too large) norms given in Eq. 2.

2 $$\:\sum\:_{i=1}^{M}\:p\left({\beta\:}_{kj};\lambda\:\right):=\alpha\:\lambda\:\sum\:_{i=1}^{M}\:{\beta\:}_{k,i}^{2}+(1-\alpha\:)\lambda\:\sum\:_{i=1}^{M}\:\left|{\beta\:}_{kj}\right|$$

#### Random Forest (RF)

RF is a highly useful ML algorithm that not only provides strong prediction results but also addresses the feature selection problem even with a larger number of related variables and contributes to model interpretability^59^. RF reveals the importance of features by considering the frequency counts of the selected features in each tree. The most selected variable is the most important feature. Another feature importance measure is the Gini index, which measures how well the samples are classified in each class. There is evidence that RF is a strong feature selection method for many types of problems^59,60^.

#### Recursive Feature Elimination (RFE)

Iterative feature elimination makes predictions with the entire feature set based on a specific learning algorithm. RFE ranks the features considering the feature’s collaboration with the main prediction model (a significant contribution to the model performance)^60^. Guyon et al. (2002) proposed RFE for feature selection in cancer prediction problems using SVM. In this proposed approach, prediction is made with all the features and the worst features are iteratively eliminated until a specified stopping criterion (a certain number of features) is met^61^.

### Prediction methods

#### Support Vector Machine (SVM)

Introduced by Vapnik (1995), SVM is a supervised learning algorithm distinguished for its ability to minimize structural risk by limiting model complexity in pattern recognition and regression problems^62,63^. It is based on statistical learning theory and optimization principles, making it a powerful tool for real-world applications in various fields such as bioinformatics, text classification, and computer vision^64^. Although the initial and basic model of SVM was developed for classifying linearly separable data, it can also map non-linearly separable data into a high-dimensional space using kernel functions, allowing for linear modeling of the data. The performance of SVM depends on the appropriate selection of hyperparameters, such as the kernel function and regularization parameter, which are determined by the user. Carefully tuning these hyper-parameters plays a crucial role in enhancing the model’s accuracy and generalization ability^65,66^.

#### Random Forest (RF)

Developed by Breiman (2001), RF is a non-linear and non-parametric ensemble learning algorithm that combines decision trees, which are weak learners, to improve prediction accuracy and stability^67^. With RF, each decision tree is trained on a random subset of the training data, and predictions are made using the majority vote of these trees. This addresses the problem of overfitting that often occurs with single decision trees, and reduces the high variance of predictions using techniques such as bootstrap. RF can be made more robust to overfitting with additional adjustments, such as adjusting the tree size, and is less sensitive to parameter tuning than other ML algorithms^68,69^.

*Extreme Gradient Boosting (XGBoost)*: XGBoost is an extension of the gradient boosting algorithm developed by Chen and Guestrin (2016) and is a high-performance, scalable ML method^70^. The objective function of XGBoost consists of two parts: the loss function and the regularization term. Gradient boosting, which forms the basis of XGBoost, minimizes the negative gradient of the loss function to adjust the parameters of each decision tree, while XGBoost uses Newton boosting for this stage. The regularization term controls the tree complexity and, thus, the overfitting error. Generally, XGBoost improves model accuracy and speed by using a sequential learning process where each new tree attempts to correct the errors of the previous trees. XGBoost runs faster compared to other ML algorithms and delivers effective results even on a single CPU. This feature provides a significant advantage when working with large datasets^71,72^.

#### Categorical Boosting (CatBoost)

CatBoost is a specialized version of the gradient boosting algorithm, optimized for categorical data. In CatBoost, a prediction model is built using a series of decision trees during training. Unlike traditional gradient boosting, ordered boosting mitigates prediction shifts by providing information to the next tree, thereby improving the model’s accuracy^73^. Since categorical features are processed during the training phase, it minimizes computational time and model complexity^74^. In addition, CatBoost is less sensitive to hyperparameter settings and generally shows high performance even with default settings^71^.

### Hyperparameter tunning

The hyperparameter tuning process was carried out within predefined search spaces for each classification model. For the SVM, the penalty parameter **C** was varied across the values [0.125, 0.5, 1, 2, 8, 10, 32, 100, 128, 512, 1000, 2048, 8192], while the kernel coefficient ***gamma*** was explored over [1e − 5, 1e − 4, 5e − 3, 2e − 3, 1e − 3, 0.01, 0.1]. These hyperparameter grids were defined using a mixed logarithmic scaling strategy, combining base-2 scaling for C and base-10 scaling for gamma, to ensure systematic and sufficiently dense exploration of the parameter space across multiple orders of magnitude. The ***radial basis function*** (RBF) kernel was employed in all cases. To avoid missing locally optimal configurations, the search grid was expanded with intermediate values, prioritizing empirical performance stability over strict logarithmic scaling.

For the RF model, the ***number of estimators*** was adjusted between [100, 200, 300, 400, 500, 800, 1000]. The ***maximum tree depth*** was tested at values [5, 10, 30, 50], whereas the ***minimum samples*** required to split a node were set to [2, 5, 10]. In addition, the ***minimum number of samples*** per leaf was considered as [1, 2, 4], with bootstrap sampling enabled (True).

For the XGBoost algorithm, the ***number of estimators*** was examined in the range [100, 200, 300, 500, 1000], with ***maximum tree depths*** of [3, 5, 10]. The ***learning rate*** was tuned over [0.001, 0.01, 0.1, 1, 10], while the ***gamma*** parameter was varied among [0.5, 1, 5].

Finally, for the CatBoost model, the ***number of estimators*** was similarly explored within [100, 200, 300, 500, 1000]. ***Maximum depths*** were considered at [1, 3, 5, 7, 9, 15], while the ***learning rate*** was varied across [0.01, 0.1, 0.5, 1]. The L2 ***leaf regularization coefficient*** was adjusted between [0.01, 0.1, 0.5].

### Evaluation metrics

The performance of the classification models was evaluated using statistical parameters derived from the confusion matrix. Specifically, the matrix consists of four fundamental components: true positives (TP), true negatives (TN), false positives (FP), and false negatives (FN). Here, TP denotes the number of toxic compounds correctly identified as toxic, whereas TN corresponds to non-toxic compounds accurately recognized as non-toxic. Conversely, FP refers to non-toxic compounds that were erroneously classified as toxic, while FN indicates toxic compounds that were incorrectly predicted as non-toxic. Based on these values, a set of performance metrics was computed, including accuracy, sensitivity, precision, the F1-Score, and the MCC, as defined in Eqs. (3)–(9)

3 $$\:\mathrm{Accuracy\:}=\frac{TP+TN}{TP+FP+TN+FN}\:\:$$

4 $$\:\mathrm{Sensitivity\:}=\frac{TP}{TP+FN}$$

5 $$\:\mathrm{Precision\:}=\frac{TP}{TP+FP}$$

6 $$\:F1-\mathrm{\:Score\:}=\frac{2\mathrm{*}TP}{2\mathrm{*}TP+FP+FN}$$

7 $$\:MCC=\frac{\left(TP\mathrm{*}TN\right)-\left(FP\mathrm{*}FN\right)}{\sqrt{(TP+FP)\mathrm{*}(TP+FN)\mathrm{*}(TN+FP)\mathrm{*}(TN+FN)}}$$

8 $$\:Cohe{n}^{{\prime\:}}s\:Kappa\,=\:\frac{{p}_{0}-{p}_{e}}{{p}_{0}-{p}_{e}}\,where,\:{p}_{0}=\frac{TP+TN}{N},\:\:{p}_{e}=\frac{\left(\mathrm{T}\mathrm{P}+\mathrm{F}\mathrm{P}\right)\left(\mathrm{T}\mathrm{P}+\mathrm{F}\mathrm{N}\right)+\left(\mathrm{F}\mathrm{N}+\mathrm{T}\mathrm{N}\right)\left(\mathrm{F}\mathrm{P}+\mathrm{T}\mathrm{N}\right)}{{N}^{2}}$$

9 $$\:ROC-AUC=\underset{0}{\overset{1}{\int\:}}TPR\left(FPR\right)d\left(FPR\right)\:where,\:TPR=\frac{TP}{TP+FN},\:\:FPR=\frac{FP}{FP+TN}$$

            

The value of these performance metrics corresponds to 1 for perfect classification and 0 for random classification.

### Applicability domain

In this study, chemical similarity was evaluated to determine the applicability domain and assess the reliability of our model. The Tanimoto similarity index is a metric used to measure chemical diversity, commonly used in chemistry and pharmaceutical research. This coefficient is widely applied for molecular similarity calculations, especially when analyzing large datasets, as it quantifies molecular resemblance based on structural properties^75^.

The extended form of the Tanimoto similarity coefficient for non-binarized data is provided in Eq. 10. This coefficient ranges between 0 and 1, where the lower and upper bounds correspond to complete dissimilarity and identical fingerprints, respectively^76^.

10 $$\:{S}_{A,B}=\frac{\sum\:_{j=1}^{j=n}\:\:{x}_{jA}{x}_{jB}}{\sum\:_{j=1}^{j=n}\:\:{\left({x}_{jA}\right)}^{2}+\sum\:_{j=1}^{j=n}\:\:{\left({x}_{jB}\right)}^{2}-\sum\:_{j=1}^{j=n}\:\:{x}_{jA}{x}_{jB}}$$

**Fig. 8:**
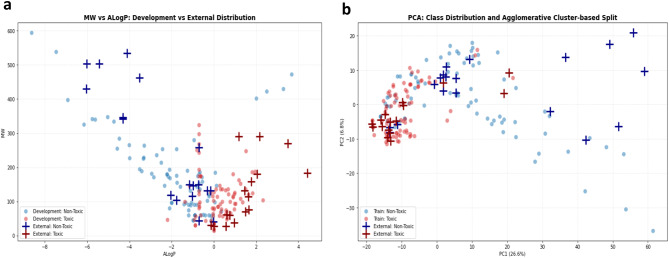
Distribution of chemical space in training and external sets**. a**, Distribution of compounds in the molecular weight (MW)–AlogP space for the development and external datasets. **b**, Principal component analysis (PCA) of the scaled descriptor space illustrating the agglomerative cluster-based split. The analytical figures were produced using Python (version 3.12.7) scripts executed in the Spyder integrated development environment under the Anaconda distribution. The official software sources are Python (https://www.python.org/), Anaconda (https://www.anaconda.com/), and Spyder (https://www.spyder-ide.org/).

The mean Tanimoto similarity scores in our study were 0.9430 for the training set, 0.8606 for the external set, and 0.6433 between the training and external sets, indicating a high level of structural similarity across datasets. To analyze chemical space distribution, the compounds in our dataset were mapped onto a multidimensional chemical space. The distribution of training and external sets within this space provides insights into the extent of chemical diversity. To further evaluate chemical diversity, molecular weight (MW) and Ghose - Crippen LogKow (AlogP) analyses were conducted for both the training and external test sets, as shown in Fig. 8a. The MW values ranged from 14.032 to 594.558, while the AlogP values varied between.

-8.62 and 4.433, demonstrating that both subsets span comparable physicochemical ranges.

In addition, principal component analysis (PCA) of the scaled descriptor space was used as a complementary qualitative assessment of chemical space coverage (Fig. 8b). While substantial overlap between the training and external sets is observed in the central regions of the PCA projection, the external set also extends into less densely populated areas of the chemical space. Taken together, the Tanimoto similarity analysis, MW–AlogP distributions, and PCA visualization were considered sufficient, within the scope of this study, to support applicability-domain consistency while allowing the use of a non-overlapping external validation set.

### SHapley Additive Explanations (SHAP)

The SHAP method, introduced as an XAI technique, aims to measure the contribution of ML model features to the output based on game theory. Shapley values, calculated based on the mathematical infrastructure of cooperative game theory, express the average marginal contribution of each feature over all possible combinations in the feature space^9^.

SHAP explanations can be computed model-agnostically to interpret both the model’s overall behavior (global) and the decisions made for a specific instance (local). Compared to other interpretability approaches such as LIME and DeepLIFT, the theoretical robustness of the SHAP method stems from its ability to satisfy three desirable properties (local accuracy, missingness, and consistency) highlighted by Lundberg and Lee (2017)^14^. Notably, SHAP is the only method that fulfills all these criteria, making it a theoretically sound approach for model interpretability.

A cooperative game is played by a set of players $\:\mathcal{M}=\left\{\mathrm{1,2},\dots\:,\:\mathcal{M}\right\}$, referred to as the grand coalition. This corresponds to a set of d-dimensional features in a prediction model. A set function $\:\nu\::\:{2}^{\mathcal{M}}⟶\mathbb{R}$ characterizes the game for all possible subsets of $\:\mathcal{M}$, such that $\:\nu\:$(*S*) represents the payoff for any coalition of players $\:S\subseteq\:\mathcal{M}\mathcal{\:}\mathcal{\:}$(which can be interpreted as the model’s loss function), with ($\:\varnothing\:$)=0.

Shapley values are derived by analyzing the marginal contribution of a player to an existing coalition *S*’ evaluated as $\:v(S\cup\:\{i\}-v\left(S\right)$for each subset *S*. The Shapley value of a player, denoted as $\:{\varphi\:}_{i}\left(v\right)\:$(Eq. 11), represents a weighted sum of its marginal contributions across all possible coalitions.

11 $$\:{\varphi\:}_{i}\left(v\right)=\sum\:_{S\subseteq\:M\backslash\:i}\frac{\left|S\right|!\left(\left|M\right|-\left|S\right|-1\right)!}{\left|M\right|!}\left[v(S\cup\:\left\{i\right\}-v\left(S\right)\right]$$

The exact calculation of SHAP values is an NP-hard problem requiring exponential time complexity. Therefore, it may not be practical in high-dimensional datasets due to the cost of computing Shapley values^77^. Another difficult aspect of the SHAP explanation is that it assumes that features are generally independent. SHAP values calculated under this assumption may reflect unrealistic effects when features are correlated^78^. To overcome some of these challenges, the use of conditional or marginal distributions can be a solution. The TreeSHAP method is significantly faster and considers feature dependencies by relying on conditional expectation^79,80^. However, this method is limited to tree-based models, and like the original approach, it still tends to assign Shapley values to features that do not influence the prediction. Therefore, by using different feature selection methods, we aim to exclude from the analysis those attributes that are not informative and may lead to misleading Shapley values due to their correlations.

### Pilot compound selection for descriptor optimization

The compound to be modified was identified through a layered selection process that combines expert opinion and the TOPSIS method, a well-established MCDM approach. TOPSIS, introduced by Hwang and Yoon (1981), is a compensatory aggregation method that ranks alternatives based on their simultaneous proximity to the positive ideal solution and distance from the negative ideal solution^81^. Its robustness in managing multiple criteria with different units and scales is particularly advantageous in environmental toxicology applications, including the comparison of chemical processes^82^, chemical process design^83^, and evaluating the quality of urban environments^84^.

## Supplementary Information

Below is the link to the electronic supplementary material.

Supplementary Material 1

Supplementary Material 2

Supplementary Material 3

## Data Availability

The analysis code and the calculated descriptor dataset supporting this study have been deposited in Supplementary File 1 in Zenodo (DOI: https://doi.org/10.5281/zenodo.19234931). The descriptor dataset is provided within the archive under the “data” directory as IndToxData.xlsx.
