# Supplementary material for: Safe-by-redesign guidance for toxic industrial chemicals using explainable artificial intelligence: Introducing the DETOX-QSAR model
Source: Sci Rep. 2026 Apr 18;16:18072. doi: 10.1038/s41598-026-48176-0 (PMC13254317; doi:10.1038/s41598-026-48176-0)
Supplement: Supplementary file 1 — Supplementary Material 1 [file 41598_2026_48176_MOESM1_ESM.docx]

**Supplementary Equation 1**

Technique for Order of Preference by Similarity to Ideal Solution (TOPSIS) method.

1. Identify evaluation criteria for alternatives.
2. Create a decision matrix ($x_{ij})$
3. Normalize the values of the evaluation criteria to make them comparable, according to the equation:

$$n_{ij}=\frac{x_{ij}}{\sqrt{\sum_{i=1}^{m} x^{2}}}$$

4. Construct a weighted normalized decision matrix:

$$v_{ij}=n_{ij}\cdot w_{j}$$

5. Determine the model coordinates of the ideal solution $\left( A^{+} \right)$and the anti-ideal solution $\left( A^{-} \right)$:

$$A^{+}=\left\{ v_{1}^{+},v_{2}^{+},\cdots,v_{m}^{+} \right\}$$

$$v_{j}^{+}=\left\{ \left( \max_{i} v_{ij}\mid j\in S \right),\left( \min_{i} v_{ij}\mid j\in D \right)\mid i=1,2,\cdots,n \right\}$$

$$A^{-}=\left\{ v_{1}^{-},v_{2}^{-},\cdots,v_{m}^{-} \right\}$$

$$v_{j}^{-}=\left\{ \left( \min_{i} v_{ij}\mid j\in S \right),\left( \max_{i} v_{ij}\mid j\in D \right)\mid i=1,2,\cdots,n \right\}$$

6. Calculate positive distance ( $D_{i}^{+}$) and a negative distance $\left( D_{i}^{-} \right)$of each evaluated alternative:

$$D_{i}^{+}=\sqrt{\sum_{j=1}^{n} \left( v_{ij}-v_{j}^{+} \right)^{2}}$$

$$D_{i}^{-}=\sqrt{\sum_{j=1}^{n} \left( v_{ij}-v_{j}^{-} \right)^{2}}$$

7. Compute the relative closeness coefficient $\left( Q_{i} \right)$ for each alternative:

$$Q_{i}=\frac{D_{i}^{-}}{D_{i}^{-}+D_{i}^{+}}$$

where: $0\leq P_{i}\leq1,i=1,2,\ldots,m$.

1. Rank the alternatives in descending order based on Qᵢ.
