# Supplementary material for: Safe-by-redesign guidance for toxic industrial chemicals using explainable artificial intelligence: Introducing the DETOX-QSAR model
Source: Sci Rep. 2026 Apr 18;16:18072. doi: 10.1038/s41598-026-48176-0 (PMC13254317; doi:10.1038/s41598-026-48176-0)
Supplement: Supplementary file 2 — Supplementary Material 2 [file 41598_2026_48176_MOESM2_ESM.docx]

**Supplementary Note 1**

**Content**

**1. Dataset Details**

1.1. Data Collection

1.2. Background of the Chemicals in the Dataset

1.2.1. Criteria for Classifying Toxic Industrial Chemicals

1.2.2. Details about Green Circle Chemicals

**1. Dataset Details**

**1.1. Data Collection**

The binary dataset (n_total_=197) included toxic and safe (non-toxic) chemicals for acute inhalation toxicity (Supplementary Table S3). The toxic chemical group consisted of all available toxic industrial chemicals (TICs) (n=98) from the Occupational Safety and Health Administration (OSHA) website^1^. Safe chemicals were gathered from “Green Circle” compounds (n=99) listed on the United States Environmental Protection Agency’s (EPA) Safer Chemical Ingredients List (SCIL)^2^. In this study, only TICs were evaluated for acute inhalation toxicity because of their potential to cause immediate lethal effects. The data on the acute inhalation toxicity risk of these compounds was verified from the European Chemicals Agency (ECHA) website, based on the Globally Harmonized System of Classification and Labeling of Chemicals (GHS) hazard codes. The globally recognized GHS Hazard Statements categorize acute inhalation toxicity into five hazard levels, described using the LCt_50_ value (Supplementary Table S1)^3,4^. Despite varying hazard levels of TICs, this study employed a binary classification approach. Specifically, the focus was on the presence of acute inhalation toxicity, not the severity levels. Therefore, all TICs (n=98) assigned any of the following H codes—H330 (n=74), H331 (n=21), H332 (n=2), and H335 (n=1)—were grouped under the label “toxic”. The 99 EPA-designated Green Circle compounds without a corresponding GHS risk category and no evidence of acute inhalation toxicity were classified as “non-toxic”. This binary classification clearly differentiates hazardous substances from safe ones based on their potential for acute inhalation toxicity.

Supplementary Table S1. Classification related to acute inhalation toxicity (based on the LCt_50_ value) according to the GHS guidelines^3,4^

| **H Code** | **Hazard Statements** | **Hazard Classification Criteria**  **(based on LCt_50_ value)** | | **Signal Words** | **Pictograms** |
| --- | --- | --- | --- | --- | --- |
| H330 | Fatal | Dusts and mists | < 0.05 mg/L | Danger | 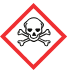 |
|  |  | Gases | < 100 ppm/V |  |  |
|  |  | Vapors | < 0.5 mg/L |  |  |
| H331 | Toxic | Dusts and mists | 0.05 - 0.5 mg/L | Danger | 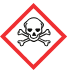 |
|  |  | Gases | 100 - 500 ppm/V |  |  |
|  |  | Vapors | 0.5 - 2.0 mg/L |  |  |
| H332 | Harmful | Dusts and mists | 0.5 - 1.0 mg/L | Danger | 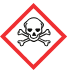 |
|  |  | Gases | 500 - 2500 ppm/V |  |  |
|  |  | Vapors | 2.0 - 10 mg/L |  |  |
| H334 | May cause allergy, asthma, or breathing issues | Dusts and mists | 1.0 - 5 mg/L | Warning | 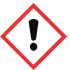 |
|  |  | Gases | 2500 - 5000 ppm/V |  |  |
|  |  | Vapors | 10 - 20 mg/L |  |  |
| H335 | May cause respiratory irritation | - | - | Warning | No Symbol |

**1.2. Background of the Chemicals in the Dataset**

**1.2.1. Criteria for Classifying Toxic Industrial Chemicals**

The Hazard Index (HI) is designed to provide a reliable risk assessment of industrial chemicals before their market launch. For an industrial chemical to be classified as a TIC, an HI must be assigned. The NATO International Task Force 25 (ITF-25) assesses the HI using four parameters (toxicity, physical state, distribution, and producer) (1), each rated on a scale of 1 to 5 (Supplementary Table S2). TICs are divided into three HI categories based on their scores: High (81 or above), Medium (36 to 80), and Low (below 36). All three categories include chemicals capable of causing acute inhalation toxicity following 30-minute exposures at low levels. The Immediately Dangerous to Life and Health (IDLH) value, used as a toxicity parameter in assessing HI, represents a critical exposure threshold. Defined by the National Institute for Occupational Safety and Health (NIOSH), IDLH indicates the airborne contaminant concentration that can cause death, serious immediate health effects (for mammals), or prevent escape from the environment. The IDLH value is calculated using the LCt50 metric, which serves as the benchmark for acute inhalation toxicity^5^.

HI = f{(toxicity)x(state)x(distribution)x(producer)} (1)

Supplementary Table S2. Hazard index (HI) parameters

| **Toxicity (IDLH in ppm)** | **Score** | **State (VP in torr)** | | | **Score** | **Distribution (CN)** | **Score** | **Producer (MN)** | **Score** |  |
| --- | --- | --- | --- | --- | --- | --- | --- | --- | --- | --- |
| IDLH<1 | 5 | Gas | |  | 5 | CN>5 | 5 | MN>100 | 5 |  |
| 1<IDLH<10 | 4 | Liquid | VP<400 | | 4 | CN=4 | 4 | 50<MN<99 | 4 |  |
| 11<IDLH<100 | 3 |  | 100<VP<400 | | 3 | CN=3 | 3 | 25<MN<49 | 3 |  |
| 101<IDLH<500 | 2 |  | 10<VP<100 | | 2 | CN=2 | 2 | 5<MN<24 | 2 |  |
| IDLH>500 | 1 |  | VP<10 | | 1 | CN=1 | 1 | MN<5 | 1 |  |
| IDLH: Immediately Dangerous to Life and Health; VP: Vapor pressure; CN: Continent number; MN: Manufacturer number | | | | | | | | | | |

**1.2.2. Details about Green Circle Chemicals**

The SCIL, developed by the EPA, encourages manufacturers to use safe ingredients to protect health and the environment. The listed chemicals are categorized into four groups based on toxicological data and environmental impact: Green Circle (lowest risk), Green Half-Circle (low risk, with potential data gaps), Yellow Triangle (limited risk), and Grey Square (not yet classified)^2^. Our acute inhalation-safe chemicals (non-toxic) were sourced from Green Circle Chemicals of the EPA, posing no significant risk of toxic effects.

# Supplementary Table S3. Dataset list of selected chemicals

| **No.** | **Name** | **CAS No** | **Hazard Statement Code(s)** | **Hazard Index** | **Molecular Formula** | **Canonical SMILES** | **training or external set** |
| --- | --- | --- | --- | --- | --- | --- | --- |
|  | 1,2-Butanediol | [584-03-2](https://www.epa.gov/saferchoice/safer-ingredients#pop584032) | - | non-toxic | [C_4_H_10_O_2_](https://pubchem.ncbi.nlm.nih.gov/#query=C4H10O2) | CCC(CO)O | training set |
|  | 1-Docosanol | 661-19-8 | - | non-toxic | [C_22_H_46_O](https://pubchem.ncbi.nlm.nih.gov/#query=C22H46O) | CCCCCCCCCCCCCCCCCCCCCCO | training set |
|  | Acetic acid | [64-19-7](https://www.epa.gov/saferchoice/safer-ingredients#pop64197) | - | non-toxic | [C_2_H_4_O_2_](https://pubchem.ncbi.nlm.nih.gov/#query=C2H4O2) | CC(=O)O | training set |
|  | Adipic acid | 124-04-9 | - | non-toxic | C_6_H_10_O_4_ | C(CCC(=O)O)CC(=O)O | external set |
|  | Alanine | 164462-16-2 | - | non-toxic | C_3_H_7_NO_2_ | CC(C(=O)O)N | training set |
|  | alpha-Methylglucoside | 97-30-3 | - | non-toxic | C_7_H_14_O_6_ | COC1C(C(C(C(O1)CO)O)O)O | training set |
|  | alpha-Tocopherol | 59-02-9 | - | non-toxic | C_29_H_50_O_2_ | CC1=C(C2=C(CCC(O2)(C)CCCC(C)CCCC(C)CCCC(C)C)C(=C1O)C)C | training set |
|  | Ammonium lauryl sulfate | 2235-54-3 | - | non-toxic | [C_12_H_29_NO_4_S](https://pubchem.ncbi.nlm.nih.gov/#query=C12H29NO4S) | CCCCCCCCCCCCOS(=O)(=O)[O-].[NH4+] | training set |
|  | Ascorbic acid | 50-81-7 | - | non-toxic | C_6_H_8_O_6_ | C(C(C1C(=C(C(=O)O1)O)O)O)O | training set |
|  | Bentonite | 1302-78-9 | - | non-toxic | Al_2_H_2_O_6_Si | O.O=[Al]O[Al]=O.O=[Si]=O | training set |
|  | Benzenepropanol | 122-97-4 | - | non-toxic | C_9_H_12_O | C1=CC=C(C=C1)CCCO | training set |
|  | Benzenesulfonic acid | [69669-44-9](https://www.epa.gov/saferchoice/safer-ingredients#pop69669449) | - | non-toxic | C_6_H_6_O_3_S | C1=CC=C(C=C1)S(=O)(=O)O | training set |
|  | Benzoic acid | 65-85-0 | - | non-toxic | C_7_H_6_O_2_ | C1=CC=C(C=C1)C(=O)O | training set |
|  | Butyl D-glucoside | 31387-97-0 | - | non-toxic | C_10_H_20_O_6_ | CCCCOC1C(C(C(C(O1)CO)O)O)O | training set |
|  | Calcium acetate | 62-54-4 | - | non-toxic | [C_4_H_6_CaO_4_](https://pubchem.ncbi.nlm.nih.gov/#query=C4H6CaO4) | CC(=O)[O-].CC(=O)[O-].[Ca+2] | training set |
|  | Calcium carbonate | 471-34-1 | - | non-toxic | CaCO_3_ | C(=O)([O-])[O-].[Ca+2] | training set |
|  | Calcium formate | 544-17-2 | - | non-toxic | C_2_H_2_CaO_4_ | C(=O)[O-].C(=O)[O-].[Ca+2] | training set |
|  | Caprylyl glycol | 1117-86-8 | - | non-toxic | [C_8_H_18_O_2_](https://pubchem.ncbi.nlm.nih.gov/#query=C8H18O2) | CCCCCCC(CO)O | training set |
|  | Decanoic acid | 334-48-5 | - | non-toxic | C_10_H_20_O_2_ | CCCCCCCCCC(=O)O | training set |
|  | delta-Tocopherol | 119-13-1 | - | non-toxic | C_27_H_46_O_2_ | CC1=CC(=CC2=C1OC(CC2)(C)CCCC(C)CCCC(C)CCCC(C)C)O | training set |
|  | Dipropylene glycol | 25265-71-8 | - | non-toxic | C_10_H_22_O_3_ | CCCCOCC(C)OCC(C)O | training set |
|  | DL-Methionine | 59-51-8 | - | non-toxic | C_5_H_11_NO_2_S | CSCCC(C(=O)O)N | external set |
|  | Docosanoic acid | 112-85-6 | - | non-toxic | C_22_H_44_O_2_ | CCCCCCCCCCCCCCCCCCCCCC(=O)O | training set |
|  | Ethanol | 64-17-5 | - | non-toxic | C_2_H_6_O | CCO | training set |
|  | Ethylene glycol dipalmitate | 624-03-3 | - | non-toxic | C_34_H_66_O_4_ | CCCCCCCCCCCCCCCC(=O)OCCOC(=O)CCCCCCCCCCCCCCC | training set |
|  | Fumaric acid | 110-17-8 | - | non-toxic | C_4_H_4_O_4_ | C(=CC(=O)O)C(=O)O | training set |
|  | Glucoheptanoic acid | 23351-51-1 | - | non-toxic | [C_7_H_14_O_8_](https://pubchem.ncbi.nlm.nih.gov/#query=C7H14O8) | C(C(C(C(C(C(C(=O)O)O)O)O)O)O)O | training set |
|  | Gluconolactone | 90-80-2 | - | non-toxic | [C_6_H_10_O_6_](https://pubchem.ncbi.nlm.nih.gov/#query=C6H10O6) | C(C1C(C(C(C(=O)O1)O)O)O)O | training set |
|  | Glycerol | 56-81-5 | - | non-toxic | C_3_H_8_O_3_ | C(C(CO)O)O | training set |
|  | Glycine | 56-40-6 | - | non-toxic | C_2_H_5_NO_2_ | C(C(=O)O)N | training set |
|  | Glycol distearate | 627-83-8 | - | non-toxic | C_38_H_74_O_4_ | CCCCCCCCCCCCCCCCCC(=O)OCCOC(=O)CCCCCCCCCCCCCCCCC | training set |
|  | Hexanoic acid | 142-62-1 | - | non-toxic | [C_6_H_12_O_2_](https://pubchem.ncbi.nlm.nih.gov/#query=C6H12O2) | CCCCCC(=O)O | external set |
|  | Hexyl D-glucoside | 54549-24-5 | - | non-toxic | C_12_H_24_O_6_ | CCCCCCOC1C(C(C(C(O1)CO)O)O)O | training set |
|  | Isopropanol | 67-63-0 | - | non-toxic | C_3_H_8_O | CC(C)O | training set |
|  | Kaolin | 1332-58-7 | - | non-toxic | [Al_2_H_4_O_9_Si_2_](https://pubchem.ncbi.nlm.nih.gov/#query=Al2H4O9Si2) | O.O.O=[Al]O[Si](=O)O[Si](=O)O[Al]=O | external set |
|  | Lauramidopropyl betaine | 4292-10-8 | - | non-toxic | C_19_H_38_N_2_O_3_ | CCCCCCCCCCCC(=O)NCCC[N+](C)(C)CC(=O)[O-] | training set |
|  | Lauryl glucoside | 27836-64-2 | - | non-toxic | [C_18_H_36_O_6_](https://pubchem.ncbi.nlm.nih.gov/#query=C18H36O6) | CCCCCCCCCCCCOC1C(C(C(C(O1)CO)O)O)O | training set |
|  | Lignin | 9005-53-2 | - | non-toxic | C_18_H_13_N_3_Na_2_O_8_S_2_ | CC(=O)NC1=C2C(=C(C=C1)S(=O)(=O)[O-])C=C(C(=C2O)N=NC3=CC=CC=C3)S(=O)(=O)[O-].[Na+].[Na+] | external set |
|  | Linoleic acid | 60-33-3 | - | non-toxic | C_18_H_32_O_2_ | CCCCCC=CCC=CCCCCCCCC(=O)O | training set |
|  | L-Lactic acid | 79-33-4 | - | non-toxic | C_3_H_6_O_3_ | CC(C(=O)O)O | training set |
|  | L-Leucine | 61-90-5 | - | non-toxic | C_6_H_13_NO_2_ | CC(C)CC(C(=O)O)N | external set |
|  | Magnesium acetate | 142-72-3 | - | non-toxic | [C_4_H_6_MgO_4_](https://pubchem.ncbi.nlm.nih.gov/#query=C4H6MgO4) | CC(=O)[O-].CC(=O)[O-].[Mg+2] | training set |
|  | Magnesium oxide | 1309-48-4 | - | non-toxic | MgO | O=[Mg] | external set |
|  | Maltodextrin | 9050-36-6 | - | non-toxic | C_18_H_32_O_16_ | C(C1C(C(C(C(O1)OC2C(OC(C(C2O)O)OC3C(OC(C(C3O)O)O)CO)CO)O)O)O)O | external set |
|  | Methyl laurate | 111-82-0 | - | non-toxic | [C_13_H_26_O_2_](https://pubchem.ncbi.nlm.nih.gov/#query=C13H26O2) | CCCCCCCCCCCC(=O)OC | training set |
|  | Nitrogen | 7727-37-9 | - | non-toxic | N_2_ | N#N | external set |
|  | Octanoic acid | 124-07-2 | - | non-toxic | C_8_H_16_O_2_ | CCCCCCCC(=O)O | training set |
|  | Palmitoleic acid | 2091-29-4 | - | non-toxic | C_16_H_30_O_2_ | CCCCCCC=CCCCCCCCC(=O)O | training set |
|  | Pentanedioic acid | 14035-94-0 | - | non-toxic | [C_5_H_8_O_4_](https://pubchem.ncbi.nlm.nih.gov/#query=C5H8O4) | C(CC(=O)O)CC(=O)O | training set |
|  | Pentaric acid | 488-31-3 | - | non-toxic | [C_5_H_8_O_7_](https://pubchem.ncbi.nlm.nih.gov/#query=C5H8O7) | C(C(C(=O)O)O)(C(C(=O)O)O)O | training set |
|  | Pentylene glycol | 5343-92-0 | - | non-toxic | [C_5_H_12_O_2_](https://pubchem.ncbi.nlm.nih.gov/#query=C5H12O2) | C(CCO)CCO | external set |
|  | Perlite | 93763-70-3 | - | non-toxic | Al_2_CaFe_2_K_2_MgNa_2_O_12_Si | [O-2].[O-2].[O-2].[O-2].[O-2].[O-2].[O-2].[O-2].O=[Mg].O=[Si]=O.O=[Ca].[Na+].[Na+].[Al+3].[Al+3].[K+].[K+].[Fe+3].[Fe+3] | training set |
|  | Poloxalene | 9003-11-6 | - | non-toxic | [C_5_H_10_O_2_](https://pubchem.ncbi.nlm.nih.gov/#query=C5H10O2) | CC1CO1.C1CO1 | training set |
|  | Polymaleic acid | [26099-09-2](https://www.epa.gov/saferchoice/safer-ingredients#pop26099092) | - | non-toxic | C_4_H_4_O_4_ | C(=CC(=O)O)C(=O)O | training set |
|  | Polypropylene | [9003-07-0](https://www.epa.gov/saferchoice/safer-ingredients#pop9003070) | - | non-toxic | C_7_H_8_O_3_ | CC=C.C1=CC(=O)OC1=O | training set |
|  | Potassium bicarbonate | 298-14-6 | - | non-toxic | [KHCO_3_](https://pubchem.ncbi.nlm.nih.gov/#query=KHCO3) | C(=O)(O)[O-].[K+] | training set |
|  | Potassium palmitate | 2624-31-9 | - | non-toxic | C_16_H_31_KO_2_ | CCCCCCCCCCCCCCCC(=O)[O-].[K+] | training set |
|  | Potassium sulfate | 7778-80-5 | - | non-toxic | K_2_SO_4_ | [O-]S(=O)(=O)[O-].[K+].[K+] | training set |
|  | Potassium xylene sulfonate | 30346-73-7 | - | non-toxic | [C_8_H_9_KO_3_S](https://pubchem.ncbi.nlm.nih.gov/#query=C8H9KO3S) | CC1=CC(=C(C=C1)S(=O)(=O)[O-])C.[K+] | training set |
|  | Propyl acetate | 109-60-4 | - | non-toxic | C_5_H_10_O_2_ | CCCOC(=O)C | training set |
|  | Propylene glycol n-butyl ether | 5131-66-8 | - | non-toxic | [C_10_H_22_O_3_](https://pubchem.ncbi.nlm.nih.gov/#query=C10H22O3) | CCCCOCC(C)OCC(C)O | training set |
|  | Rhamnopyranosyl-3-hydroxydecanoyl-3-hydroxydecanoate | 37134-61-5 | - | non-toxic | C_26_H_48_O_9_ | CCCCCCCC(CC(=O)O)OC(=O)CC(CCCCCCC)OC1C(C(C(C(O1)C)O)O)O | external set |
|  | Silanamine | [68909-20-6](https://www.epa.gov/saferchoice/safer-ingredients#pop68909206) | - | non-toxic | H_2_NSi | N[Si] | external set |
|  | Sodium (Z)-hexadec-9-enoate | 6610-24-8 | - | non-toxic | C_16_H_29_NaO_2_ | CCCCCCC=CCCCCCCCC(=O)[O-].[Na+] | training set |
|  | Sodium acetate | 127-09-3 | - | non-toxic | C_2_H_3_NaO_2_ | CC(=O)[O-].[Na+] | training set |
|  | Sodium acetate trihydrate | 6131-90-4 | - | non-toxic | C_2_H_9_NaO_5_ | CC(=O)[O-].O.O.O.[Na+] | training set |
|  | Sodium bisulfate | 7681-38-1 | - | non-toxic | HNaSO_4_ | OS(=O)(=O)[O-].[Na+] | training set |
|  | Sodium citrate | 994-36-5 | - | non-toxic | C_6_H_5_Na_3_O_7_ | C(C(=O)[O-])C(CC(=O)[O-])(C(=O)[O-])O.[Na+].[Na+].[Na+] | training set |
|  | Sodium decanoate | 1002-62-6 | - | non-toxic | [C_10_H_19_NaO_2_](https://pubchem.ncbi.nlm.nih.gov/#query=C10H19NaO2) | CCCCCCCCCC(=O)[O-].[Na+] | training set |
|  | Sodium formate | 141-53-7 | - | non-toxic | [CHNaO_2_](https://pubchem.ncbi.nlm.nih.gov/#query=CHNaO2) | C(=O)[O-].[Na+] | training set |
|  | Sodium myristate | 822-12-8 | - | non-toxic | C_14_H_27_NaO_2_ | CCCCCCCCCCCCCC(=O)[O-].[Na+] | training set |
|  | Sodium sulfate | 7757-82-6 | - | non-toxic | Na_2_SO_4_ | [O-]S(=O)(=O)[O-].[Na+].[Na+] | training set |
|  | Sodium sulfite | 7757-83-7 | - | non-toxic | Na_2_SO_3_ | [O-]S(=O)[O-].[Na+].[Na+] | training set |
|  | Sodium toluenesulfonate | 12068-03-0 | - | non-toxic | [C_7_H_7_NaO_3_S](https://pubchem.ncbi.nlm.nih.gov/#query=C7H7NaO3S) | CC1=CC=C(C=C1)S(=O)(=O)[O-].[Na+] | training set |
|  | Sorbitan monolaurate | 1338-39-2 | - | non-toxic | [C_18_H_34_O_6_](https://pubchem.ncbi.nlm.nih.gov/#query=C18H34O6) | CCCCCCCCCCCC(=O)OCC(C1C(C(CO1)O)O)O | external set |
|  | Sorbitan monostearate | 1338-41-6 | - | non-toxic | [C_24_H_46_O_6_](https://pubchem.ncbi.nlm.nih.gov/#query=C24H46O6) | CCCCCCCCCCCCCCCCCC(=O)OCC(C1C(C(CO1)O)O)O | external set |
|  | Sorbitol | 50-70-4 | - | non-toxic | C_6_H_14_O_6_ | C(C(C(C(C(CO)O)O)O)O)O | training set |
|  | Squalane | 111-01-3 | - | non-toxic | [C_30_H_62_](https://pubchem.ncbi.nlm.nih.gov/#query=C30H62) | CC(C)CCCC(C)CCCC(C)CCCCC(C)CCCC(C)CCCC(C)C | training set |
|  | Stearic acid | 57-11-4 | - | non-toxic | C_18_H_36_O_2_ | CCCCCCCCCCCCCCCCCC(=O)O | training set |
|  | Sucrose | 57-50-1 | - | non-toxic | [C_12_H_22_O_11_](https://pubchem.ncbi.nlm.nih.gov/#query=C12H22O11) | C(C1C(C(C(C(O1)OC2(C(C(C(O2)CO)O)O)CO)O)O)O)O | external set |
|  | Sulfamic acid | [5329-14-6](https://www.epa.gov/saferchoice/safer-ingredients#pop5329146) | - | non-toxic | [H_3_NO_3_S](https://pubchem.ncbi.nlm.nih.gov/#query=H3NO3S) | NS(=O)(=O)O | training set |
|  | Tetradecanoic acid | 544-63-8 | - | non-toxic | C_14_H_28_O_2_ | CCCCCCCCCCCCCC(=O)O | training set |
|  | Tocopherol acetate | 7695-91-2 | - | non-toxic | [C_31_H_52_O_3_](https://pubchem.ncbi.nlm.nih.gov/#query=C31H52O3) | CC1=C(C(=C(C2=C1OC(CC2)(C)CCCC(C)CCCC(C)CCCC(C)C)C)OC(=O)C)C | training set |
|  | Triethylene glycol | 112-27-6 | - | non-toxic | [C_6_H_14_O_4_](https://pubchem.ncbi.nlm.nih.gov/#query=C6H14O4) | C(COCCOCCO)O | external set |
|  | Undecyl-D-glucoside | 98283-67-1 | - | non-toxic | [C_17_H_34_O_6_](https://pubchem.ncbi.nlm.nih.gov/#query=C17H34O6) | CCCCCCCCCCCOC1C(C(C(C(O1)CO)O)O)O | training set |
|  | Urea | 57-13-6 | - | non-toxic | [CH_4_N_2_O](https://pubchem.ncbi.nlm.nih.gov/#query=CH4N2O) | C(=O)(N)N | training set |
|  | Vanillin | 121-33-5 | - | non-toxic | [C_8_H_8_O_3_](https://pubchem.ncbi.nlm.nih.gov/#query=C8H8O3) | COC1=C(C=CC(=C1)C=O)O | training set |
|  | Xanthan gum | 11138-66-2 | - | non-toxic | [C_8_H_14_Cl_2_N_2_O_2_](https://pubchem.ncbi.nlm.nih.gov/#query=C8H14Cl2N2O2) | C1=CC(=C(C=C1N)N)OCCO.Cl.Cl | training set |
|  | 1,6-Hexanediol | 629-11-8 | - | non-toxic | [C_6_H_14_O_2_](https://pubchem.ncbi.nlm.nih.gov/#query=C6H14O2) | C(CCCO)CCO | external set |
|  | 1-Propanaminium, N-(carboxymethyl)-N,N-dimethyl-3-[(1-oxohexadecyl)amino]-, inner salt | 32954-43-1 | - | non-toxic | [C_23_H_46_N_2_O_3_](https://pubchem.ncbi.nlm.nih.gov/#query=C23H46N2O3) | CCCCCCCCCCCCCCCC(=O)NCCC[N+](C)(C)CC(=O)[O-] | training set |
|  | 2,3-Butanediol | 513-85-9 | - | non-toxic | [C_4_H_10_O_2_](https://pubchem.ncbi.nlm.nih.gov/#query=C4H10O2) | CC(C(C)O)O | training set |
|  | 2-Propenoic acid, 2-methyl-, polymer with butyl 2-propenoate | 25035-82-9 | - | non-toxic | C_11_H_18_O_4_ | CCCCOC(=O)C=C.CC(=C)C(O)=O | training set |
|  | 2-Propenoic acid, telomer with sodium sulfite (11), potassium salt | 91144-30-8 | - | non-toxic | [C_3_H_5_KNaO_5_S^+^](https://pubchem.ncbi.nlm.nih.gov/#query=C3H5KNaO5S+) | C=CC(=O)O.OS(=O)[O-].[Na+].[K+] | training set |
|  | Alcohols, C9-11-iso-, C10-rich, ethoxylated_319290845 | 78330-20-8 | - | non-toxic | C_12_H_26O_ | CCCCCC[C@@H](OCC)C(C)C | training set |
|  | Amyl propionate | 624-54-4 | - | non-toxic | C_8_H_16_O_2_ | CCCCCOC(=O)CC | training set |
|  | Butanoic acid, 3-hydroxy-, ethyl ester | 5405-41-4 | - | non-toxic | C_6_H_12_O_3_ | CCOC(=O)CC(C)O | external set |
|  | Butyl-3-hydroxy-2-methylbuyrate | 92011-00-2 | - | non-toxic | C_9_H_18_O_3_ | CCCCOC(=O)C(C)C(C)O | training set |
|  | C.I. Acid Red 18, trisodium salt | 2611-82-7 | - | non-toxic | [C_20_H_11_N_2_Na_3_O_10_S_3_](https://pubchem.ncbi.nlm.nih.gov/#query=C20H11N2Na3O10S3) | C1=CC=C2C(=C1)C(=CC=C2S(=O)(=O)[O-])N=NC3=C(C=CC4=CC(=CC(=C43)S(=O)(=O)[O-])S(=O)(=O)[O-])O.[Na+].[Na+].[Na+] | external set |
|  | Calcium gluconate | 299-28-5 | - | non-toxic | C_12_H_22_CaO_14_ | C([C@H]([C@H]([C@@H]([C@H](C(=O)[O-])O)O)O)O)O.C([C@H]([C@H]([C@@H]([C@H](C(=O)[O-])O)O)O)O)O.[Ca+2] | training set |
|  | Allyl isothiocyanate | 57-06-7 | H330 | Low | [C_4_H_5_NS](https://pubchem.ncbi.nlm.nih.gov/#query=C4H5NS) | C=CCN=C=S | training set |
|  | Bromine | 7726-95-6 | H330 | Low | Br_2_ | BrBr | external set |
|  | Bromine pentafluoride | 7789-30-2 | H330 | Low | BrF_5_ | FBr(F)(F)(F)F | training set |
|  | Bromine trifluoride | 7787-71-5 | H330 | Low | BrF_3_ | FBr(F)F | training set |
|  | Carbonyl fluoride | 353-50-4 | H330 | Low | [CF_2_O](https://pubchem.ncbi.nlm.nih.gov/#query=CF2O) | C(=O)(F)F | training set |
|  | Chlorine pentafluoride | 13637-63-3 | H330 | Low | [ClF_5_](https://pubchem.ncbi.nlm.nih.gov/#query=ClF5) | FCl(F)(F)(F)F | training set |
|  | Chlorine trifluoride | 7790-91-2 | H330 | Low | [ClF_3_](https://pubchem.ncbi.nlm.nih.gov/#query=ClF3) | FCl(F)F | training set |
|  | Chloroacetaldehyde | 107-20-0 | H330 | Low | C_2_H_3_ClO | C(C=O)Cl | training set |
|  | Crotonaldehyde | 123-73-9 | H330 | Low | C_4_H_6_O | CC=CC=O | training set |
|  | Cyanogen chloride | 506-77-4 | H330 | Low | [ClCN](https://pubchem.ncbi.nlm.nih.gov/#query=ClCN) | C(#N)Cl | external set |
|  | Dimethyl sulfate | 77-78-1 | H330 | Low | C_2_H_6_O_4_S | COS(=O)(=O)OC | training set |
|  | Ethyl chloroformate | 541-41-3 | H330 | Low | C_3_H_5_ClO_2_ | CCOC(=O)Cl | training set |
|  | Ethyl chlorothioformate | 2941-64-2 | H330 | Low | [C_3_H_5_ClOS](https://pubchem.ncbi.nlm.nih.gov/#query=C3H5ClOS) | CCSC(=O)Cl | training set |
|  | Ethyl phosphonothioic dichloride | 993-43-1 | H330 | Low | [C_2_H_5_Cl_2_PS](https://pubchem.ncbi.nlm.nih.gov/#query=C2H5Cl2PS) | CCP(=S)(Cl)Cl | training set |
|  | Ethyleneimine | 151-56-4 | H330 | Low | C_2_H_5_N | C1CN1 | training set |
|  | Hexachlorocyclopentadiene | 77-47-4 | H330 | Low | C_5_Cl_6_ | C1(=C(C(C(=C1Cl)Cl)(Cl)Cl)Cl)Cl | external set |
|  | Iron pentacarbonyl | 13463-40-6 | H330 | Low | [C_5_FeO_5_](https://pubchem.ncbi.nlm.nih.gov/#query=C5FeO5) | C=O.C=O.C=O.C=O.C=O.[Fe] | training set |
|  | Isopropyl chloroformate | 108-23-6 | H330 | Low | C_4_H_7_ClO_2_ | CC(C)OC(=O)Cl | training set |
|  | Isopropyl isocyanate | 1795-48-8 | H330 | Low | C_4_H_7_NO | CC(C)N=C=O | training set |
|  | n-Butyl chloroformate | 592-34-7 | H330 | Low | C_5_H_9_ClO_2_ | CCCCOC(=O)Cl | training set |
|  | n-Butyl isocyanate | 111-36-4 | H330 | Low | C_5_H_9_NO | CCCCN=C=O | training set |
|  | Nitric oxide | 10102-43-9 | H330 | Low | NO | [N]=O | external set |
|  | n-Propyl chloroformate | 109-61-5 | H330 | Low | C_4_H_7_ClO_2_ | CCCOC(=O)Cl | training set |
|  | Parathion | 56-38-2 | H330 | Low | [C_10_H_14_NO_5_PS](https://pubchem.ncbi.nlm.nih.gov/#query=C10H14NO5PS) | CCOP(=S)(OCC)OC1=CC=C(C=C1)[N+](=O)[O-] | external set |
|  | Perchloromethyl mercaptan | 594-42-3 | H330 | Low | CCl_4_S | C(SCl)(Cl)(Cl)Cl | external set |
|  | sec-Butyl chloroformate | 17462-58-7 | H330 | Low | [C_5_H_9_ClO_2_](https://pubchem.ncbi.nlm.nih.gov/#query=C5H9ClO2) | CCC(C)OC(=O)Cl | training set |
|  | tert-Butyl isocyanate | 1609-86-5 | H330 | Low | C_5_H_9_NO | CC(C)(C)N=C=O | training set |
|  | Tetraethyl lead | 78-00-2 | H330 | Low | C_8_H_20_Pb | CC[Pb](CC)(CC)CC | training set |
|  | Tetramethyl lead | 75-74-1 | H330 | Low | C_4_H_12_Pb | C[Pb](C)(C)C | training set |
|  | Toluene 2,4-diisocyanate | 584-84-9 | H330 | Low | [C_9_H_6_N_2_O_2_](https://pubchem.ncbi.nlm.nih.gov/#query=C9H6N2O2) | CC1=C(C=C(C=C1)N=C=O)N=C=O | training set |
|  | Toluene 2,6-diisocyanate | 91-08-7 | H330 | Low | [C_9_H_6_N_2_O_2_](https://pubchem.ncbi.nlm.nih.gov/#query=C9H6N2O2) | CC1=C(C=CC=C1N=C=O)N=C=O | training set |
|  | Acetone cyanohydrin | 75-86-5 | H330 | Medium | [C_4_H_7_NO](https://pubchem.ncbi.nlm.nih.gov/#query=C4H7NO) | CC(C)(C#N)O | training set |
|  | Acrolein | 107-02-8 | H330 | Medium | C_3_H_4_O | C=CC=O | training set |
|  | Allyl alcohol | 107-18-6 | H330 | Medium | [C_3_H_6_O](https://pubchem.ncbi.nlm.nih.gov/#query=C3H6O) | C=CCO | training set |
|  | Allylamine | 107-11-9 | H330 | Medium | C_3_H_7_N | C=CCN | training set |
|  | Allyl chlorocarbonate | 2937-50-0 | H330 | Medium | [C_4_H_5_ClO_2_](https://pubchem.ncbi.nlm.nih.gov/#query=C4H5ClO2) | C=CCOC(=O)Cl | training set |
|  | Boron tribromide | 10294-33-4 | H330 | Medium | BBr_3_ | B(Br)(Br)Br | training set |
|  | Chloroacetone | 78-95-5 | H330 | Medium | C_3_H_5_ClO | CC(=O)CCl | training set |
|  | Chlorosulfonic acid | 7790-94-5 | H330 | Medium | ClHO_3_S | OS(=O)(=O)Cl | training set |
|  | Diketene | 674-82-8 | H330 | Medium | C_4_H_4_O_2_ | C=C1CC(=O)O1 | training set |
|  | Hydrogen selenide | 7783-07-5 | H330 | Medium | [H_2_Se](https://pubchem.ncbi.nlm.nih.gov/#query=H2Se) | [SeH2] | training set |
|  | Methanesulfonyl chloride | 124-63-0 | H330 | Medium | CH_3_ClO_2_S | CS(=O)(=O)Cl | training set |
|  | Methyl bromide | 74-83-9 | H330 | Medium | [CH_3_Br](https://pubchem.ncbi.nlm.nih.gov/#query=CH3Br) | CBr | training set |
|  | Methyl chloroformate | 79-22-1 | H330 | Medium | [C_2_H_3_ClO_2_](https://pubchem.ncbi.nlm.nih.gov/#query=C2H3ClO2) | COC(=O)Cl | training set |
|  | Methyl hydrazine | 60-34-4 | H330 | Medium | [CH_6_N_2_](https://pubchem.ncbi.nlm.nih.gov/#query=CH6N2) | CNN | training set |
|  | Methyl isocyanate | 624-83-9 | H330 | Medium | C_2_H_3_NO | CN=C=O | training set |
|  | Nitrogen dioxide | 10102-44-0 | H330 | Medium | NO_2_ | N(=O)[O] | training set |
|  | Phosphine | 7803-51-2 | H330 | Medium | [H_3_P](https://pubchem.ncbi.nlm.nih.gov/#query=H3P) | P | training set |
|  | Phosphorus oxychloride | 10025-87-3 | H330 | Medium | Cl_3_OP | O=P(Cl)(Cl)Cl | training set |
|  | Phosphorus pentafluoride | 7647-19-0 | H330 | Medium | [F_5_P](https://pubchem.ncbi.nlm.nih.gov/#query=F5P) | FP(F)(F)(F)F | training set |
|  | Selenium hexafluoride | 7783-79-1 | H330 | Medium | F_6_Se | F[Se](F)(F)(F)(F)F | training set |
|  | Silicon tetrafluoride | 7783-61-1 | H330 | Medium | F_4_Si | F[Si](F)(F)F | training set |
|  | Stibine | 7803-52-3 | H330 | Medium | H_3_Sb | [SbH3] | training set |
|  | Sulfur trioxide | 7446-11-9 | H330 | Medium | [O_3_S](https://pubchem.ncbi.nlm.nih.gov/#query=O3S) | O=S(=O)=O | training set |
|  | Sulfuryl chloride | 7791-25-5 | H330 | Medium | [Cl_2_O_2_S](https://pubchem.ncbi.nlm.nih.gov/#query=Cl2O2S) | O=S(=O)(Cl)Cl | training set |
|  | Sulfuryl fluoride | 2699-79-8 | H330 | Medium | [F_2_SO_2_](https://pubchem.ncbi.nlm.nih.gov/#query=F2SO2) | O=S(=O)(F)F | training set |
|  | Tellurium hexafluoride | 7783-80-4 | H330 | Medium | F_6_Te | F[Te](F)(F)(F)(F)F | training set |
|  | n-Octyl mercaptan | 111-88-6 | H330 | Medium | C_8_H_18_S | CCCCCCCCS | training set |
|  | Titanium tetrachloride | 7550-45-0 | H330 | Medium | Cl_4_Ti | Cl[Ti](Cl)(Cl)Cl | training set |
|  | Trichloroacetyl chloride | 76-02-8 | H330 | Medium | [C_2_Cl_4_O](https://pubchem.ncbi.nlm.nih.gov/#query=C2Cl4O) | C(=O)(C(Cl)(Cl)Cl)Cl | external set |
|  | Trifluoroacetyl chloride | 354-32-5 | H330 | Medium | [C_2_ClF_3_O](https://pubchem.ncbi.nlm.nih.gov/#query=C2ClF3O) | C(=O)(C(F)(F)F)Cl | external set |
|  | Arsine | 7784-42-1 | H330 | High | AsH_3_ | [AsH3] | training set |
|  | Boron trichloride | 10294-34-5 | H330 | High | BCl_3_ | B(Cl)(Cl)Cl | training set |
|  | Boron trifluoride | 7637-07-2 | H330 | High | BF_3_ | B(F)(F)F | training set |
|  | Chlorine | 7782-50-5 | H330 | High | Cl_2_ | ClCl | external set |
|  | Diborane | 19287-45-7 | H330 | High | B_2_H_6_ | B.B | training set |
|  | Fluorine | 7782-41-4 | H330 | High | [F_2_](https://pubchem.ncbi.nlm.nih.gov/#query=F2) | FF | external set |
|  | Formaldehyde | 50-00-0 | H330 | High | [CH_2_O](https://pubchem.ncbi.nlm.nih.gov/#query=CH2O) | C=O | training set |
|  | Hydrogen cyanide | 74-90-8 | H330 | High | [CHN](https://pubchem.ncbi.nlm.nih.gov/#query=CHN) | C#N | external set |
|  | Hydrogen fluoride | 7664-39-3 | H330 | High | FH | F | training set |
|  | Hydrogen sulfide | 7783-0604 | H330 | High | H_2_S | S | training set |
|  | Phosgene | 75-44-5 | H330 | High | CCl_2_O | C(=O)(Cl)Cl | training set |
|  | Phosphorus trichloride | 7719-12-2 | H330 | High | Cl_3_P | P(Cl)(Cl)Cl | training set |
|  | Tungsten hexafluoride | 7783-82-6 | H330 | High | F_6_W | F[W](F)(F)(F)(F)F | training set |
|  | Arsenic trichloride | 7784-34-1 | H331 | Low | AsCl_3_ | Cl[As](Cl)Cl | training set |
|  | Bromine chloride | 13863-41-7 | H331 | Low | BrCl | ClBr | external set |
|  | Chloroacetyl chloride | 79-04-9 | H331 | Low | C_2_H_2_Cl_2_O | C(C(=O)Cl)Cl | training set |
|  | Ethyl phosphonic dichloride | 1066-50-8 | H331 | Low | C_2_H_5_Cl_2_OP | CCP(=O)(Cl)Cl | training set |
|  | Hydrogen iodide | 10034-85-2 | H331 | Low | HI | I | training set |
|  | Isobutyl chloroformate | 543-27-1 | H331 | Low | C_5_H_9_ClO_2_ | CC(C)COC(=O)Cl | training set |
|  | Acrylonitrile | 107-13-l | H331 | Medium | [C_3_H_3_N](https://pubchem.ncbi.nlm.nih.gov/#query=C3H3N) | C=CC#N | training set |
|  | Carbon monoxide | 630-08-0 | H331 | Medium | CO | [C-]#[O+] | external set |
|  | Carbonyl sulfide | 463-58-1 | H331 | Medium | [COS](https://pubchem.ncbi.nlm.nih.gov/#query=COS) | C(=O)=S | external set |
|  | Chloroacetonitrile | 7790-94-5 | H331 | Medium | [C_2_H_2_ClN](https://pubchem.ncbi.nlm.nih.gov/#query=C2H2ClN) | C(C#N)Cl | training set |
|  | 1,2-Dimethylhydrazine | 540-73-8 | H331 | Medium | C_2_H_8_N_2_ | CNNC | training set |
|  | Ethylene dibromide | 106-93-4 | H331 | Medium | C_2_H_4_Br_2_ | C(CBr)Br | training set |
|  | Methylchlorosilane | 993-00-0 | H331 | Medium | CH_5_ClSi | C[SiH2]Cl | training set |
|  | Methyl mercaptan | 74-93-1 | H331 | Medium | CH_4_S | CS | training set |
|  | Ammonia | 7664-41-7 | H331 | High | H_3_N | N | training set |
|  | Ethylene oxide | 75-21-8 | H331 | High | C_2_H_4_O | C1CO1 | training set |
|  | Hydrogen bromide | 10035-10-6 | H331 | High | BrH | Br | training set |
|  | Hydrogen chloride | 7647-01-0 | H331 | High | [ClH](https://pubchem.ncbi.nlm.nih.gov/#query=ClH) | Cl | training set |
|  | Nitric acid | 7697-37-2 | H331 | High | HNO_3_ | [N+](=O)(O)[O-] | training set |
|  | Sulfur dioxide | 7446-09-5 | H331 | High | [SO_2_](https://pubchem.ncbi.nlm.nih.gov/#query=SO2) | O=S=O | training set |
|  | Sulfuric acid | 7664-93-9 | H331 | High | H_2_O_4_S | OS(=O)(=O)O | training set |
|  | Diphenylmethane-4.4'-diisocyanate | 101-68-8 | H332 | Low | [C_15_H_10_N_2_O_2_](https://pubchem.ncbi.nlm.nih.gov/#query=C15H10N2O2) | C1=CC(=CC=C1CC2=CC=C(C=C2)N=C=O)N=C=O | training set |
|  | Carbon disulfide | 75-15-0 | H332 | High | CS_2_ | C(=S)=S | external set |
|  | Tetraethyl pyrophosphate | 107-49-3 | H335 | Low | [C_8_H_20_O_7_P_2_](https://pubchem.ncbi.nlm.nih.gov/#query=C8H20O7P2) | CCOP(=O)(OCC)OP(=O)(OCC)OCC | external set |

The safe category is based on the United States Environmental Protection Agency (EPA). Low, Medium, and High categories are based on the Toxic Industrial Chemicals (TICs) Guide, Occupational Safety and Health Administration (OSHA). Hazard Statement Code(s) are based on the European Chemicals Agency (ECHA).

**References**

1. Occupational Safety and Health Administration (OSHA), 2024. Safety and Health Guides. Toxic Industrial Chemicals (TICs) Guide. <https://www.osha.gov/emergency-preparedness/guides/toxic-industrial-chemicals> (2024).
2. United States Environmental Protection Agency (EPA). Safer Chemical Ingredients List. <https://www.epa.gov/saferchoice/safer-ingredients#scil> (2024).
3. United States Environmental Protection Agency (EPA). Chemical Hazard Classification and Labeling: Comparison of OPP Requirements and The GHS Contents. <https://www.epa.gov/sites/default/files/2015-09/documents/ghscriteria-summary.pdf> (2015).
4. European Chemicals Agency (ECHA). <https://www.echa.europa.eu/> (2024).
5. National Institute for Occupational Safety and Health (NIOSH). Derivation of Immediately Dangerous to Life or Health (IDLH) Values. <https://www.cdc.gov/niosh/docs/2014-100/pdfs/2014-100.pdf> (2014).
6. Stuempfle, A. K., Howells, D. J., Armour, S. J. & Boulet, C.A. International Task Force 25: Hazard from Industrial Chemicals Final Report. (Edgewood Research Development and Engineering Center, Aberdeen Proving Ground, 1998).
